# Standardized Nanomechanical Atomic Force Microscopy Procedure (SNAP) for Measuring Soft and Biological Samples

**DOI:** 10.1038/s41598-017-05383-0

**Published:** 2017-07-11

**Authors:** Hermann Schillers, Carmela Rianna, Jens Schäpe, Tomas Luque, Holger Doschke, Mike Wälte, Juan José Uriarte, Noelia Campillo, Georgios P. A. Michanetzis, Justyna Bobrowska, Andra Dumitru, Elena T. Herruzo, Simone Bovio, Pierre Parot, Massimiliano Galluzzi, Alessandro Podestà, Luca Puricelli, Simon Scheuring, Yannis Missirlis, Ricardo Garcia, Michael Odorico, Jean-Marie Teulon, Frank Lafont, Malgorzata Lekka, Felix Rico, Annafrancesca Rigato, Jean-Luc Pellequer, Hans Oberleithner, Daniel Navajas, Manfred Radmacher

**Affiliations:** 1 Institute of Physiology II, University of Münster, 48149 Münster, Germany; 2 Institute of Biophysics, University of Bremen, 28359 Bremen, Germany; 3 Institute for Bioengineering of Catalonia, University of Barcelona, and CIBER Enfermedades Respiratorias, 08028 Barcelona, Spain; 4 Department of Mechanical Engineering & Aeronautics, University of Patras, 265 04 Patras, Greece; 5 Institute of Nuclear Physics, Polish Academy of Sciences, PL-31342 Krakow, Poland; 6 Instituto de Ciencia de Materiales de Madrid, CSIC, Sor Juana Ines de la Cruz 3, 28049 Madrid, Spain; 7 CMPI-CIIL, CNRS UMR 8204 - INSERM U1019, Institut Pasteur de Lille - Univ Lille, F-59019 Lille, Cedex France; 8 BIAM, CEA, Aix-Marseille Univ., Saint-Paul-Lez-Durance, 13108 France; 9 CEA Marcoule, iBEB, Department of Biochemistry and Nuclear Toxicology, F-30207 Bagnols-sur-Cèze, France; 10 CIMaINa and Department of Physics, Università degli Studi di Milano, via Celoria 16, 20133 Milano, Italy; 11 College of Materials Science and Engineering, Shenzhen Key Laboratory of Polymer Science and Technology, Guangdong Research Center for Interfacial Engineering of Functional Materials, Nanshan District Key Lab for Biopolymers and Safety Evaluation, Shenzhen University, Shenzhen, 518060 PR China; 12 College of Optoelectronic Engineering, Key Laboratory of Optoelectronic Devices and System of Ministry of Education and Guangdong Province, Shenzhen University, Shenzhen, 518060 PR China; 13 U1006 INSERM, Aix-Marseille Université, Parc Scientifique et Technologique de Luminy, 13009 Marseille, France; 14 Department of Physiology and Biophysics, Weill Cornell Medicine, New York, NY 10065 USA; 15 Department of Anesthesiology, Weill Cornell Medicine, New York, NY 10065 USA; 16 ICSM, UMR 5257, CEA, CNRS, ENSCM, Univ. Montpellier, Site de Marcoule, Bât. 426, BP 17171, 30207 Bagnols-sur-Cèze, France; 17 Univ. Grenoble Alpes, CEA, CNRS, IBS, F-38000 Grenoble, France

## Abstract

We present a procedure that allows a reliable determination of the elastic (Young’s) modulus of soft samples, including living cells, by atomic force microscopy (AFM). The standardized nanomechanical AFM procedure (SNAP) ensures the precise adjustment of the AFM optical lever system, a prerequisite for all kinds of force spectroscopy methods, to obtain reliable values independent of the instrument, laboratory and operator. Measurements of soft hydrogel samples with a well-defined elastic modulus using different AFMs revealed that the uncertainties in the determination of the deflection sensitivity and subsequently cantilever’s spring constant were the main sources of error. SNAP eliminates those errors by calculating the correct deflection sensitivity based on spring constants determined with a vibrometer. The procedure was validated within a large network of European laboratories by measuring the elastic properties of gels and living cells, showing that its application reduces the variability in elastic moduli of hydrogels down to 1%, and increased the consistency of living cells elasticity measurements by a factor of two. The high reproducibility of elasticity measurements provided by SNAP could improve significantly the applicability of cell mechanics as a quantitative marker to discriminate between cell types and conditions.

## Introduction

Elasticity represents a biophysical property of a cell that influences and is influenced by physiological and pathological processes of the cell itself and its surrounding environment^1–3^. It is an integrative property summarizing the biophysical outcome of many known and unknown cellular processes. This includes intracellular signalling, cytoskeletal activity, organelle dynamics, changes of cell volume and morphology and many others. Not only do intracellular processes define cell mechanical properties, but also environmental factors like their biochemical and biophysical surrounding^4^. Thus, cell mechanics represents a comprehensive variable of life. Cell elasticity and its alterations were increasingly used during the last decades as a quantitative marker to describe the state and phenotype of cells^5–7^. Several techniques have been used to study cell mechanics and underlying mechanisms, such as optical tweezers^8^, magnetic twisting cytometry^9^, micropipette aspiration^10^ or optical stretcher technique^11^. Among all, Atomic Force Microscopy (AFM) combines precise spatial resolution with high force sensitivity allowing the investigation of mechanical properties of living adherent cells in a unique fashion^12–14^.

Elastic moduli of a large variety of cells have been investigated by AFM by many research groups in different laboratories. However, comparing measured values revealed a large variation of Young’s or elastic moduli even for the same cell type^15^. These variations can be related to two main sources: biological variability and technical inaccuracy. Single living cells, even within the same sample, show intrinsic biophysical inhomogeneity due to natural biological distributions of cell physiological activity and morphology. Also differences in cell handling, e.g. different protocols for cell isolation and preparation as well as cell culture and measurement conditions, contribute to the broad distribution of results.

The technical causes of the variability in elasticity numbers are either due to instrumental errors or to differences in protocols used for acquiring and analysing data. Instrumental errors are mainly caused by the inaccuracies in determining the deflection sensitivity, spring constant or tip geometry. Data acquisition influences the results through distinct experimental conditions, e.g. various tip velocity^16^, maximum loading force or sample position (e.g. cell central body or periphery)^17^. Data analysis contributes in affecting the final results as well, e.g. due to choice of the theoretical model applied in data processing.

The capability to obtain consistent and reproducible results, independently from operators, setups and locations would definitely help to establish AFM use on a large scale for clinical or medical applications, for example to potentially use cell mechanics as a marker to detect diseased cells^18–20^. To identify and eliminate all technical errors, a systematic standardization activity involving several laboratories, instruments and operators is mandatory to overcome the inherent limitations of single-laboratory studies. The presented study was performed within a large network of European laboratories, the *European Network on Applications of Atomic Force Microscopy to Nanomedicine and Lifesciences* (*COST Action TD 1002 AFM4NanoMed&Bio*)^21^. By taking full advantage of the variety of instruments, operators and experimental configurations provided by the network, a novel procedure was developed and validated, aimed to standardize mechanical measurements of soft and biological samples.

To identify the main instrumental error source, a preliminary set of experiments was performed on soft polyacrylamide (PA) gels in 11 different labs. Gels were produced and measured in one location and distributed among the other labs. We could observe that both the calibration of the deflection sensitivity and the force constant from the thermal fluctuations of the free cantilever are largely error-prone (see supporting informations 1 and 2). All commercial AFMs use an optical lever system to measure the bending of the cantilever^22^. A laser beam is reflected from the very end of the cantilever towards a position sensitive photodiode. For sake of rigorousness, the optical lever method does not measure the bending of the cantilever itself but its bending angle. In addition, the position sensitive photodiode will have a linear response curve only for small signal changes. In the linear regime, the sensitivity of the deflection signal needs to be calibrated by an appropriate procedure. In the conventional procedure, a force curve is recorded in a bare region of a rigid substrate and a linear fit is done in some regime of this data to obtain a conversion factor converting the voltage measured from the sensor to deflection signal (presented in nm/V). This conversion factor is most often called *deflection sensitivity*, although other names are also used (e.g. InvOLS: Inverse Optical Lever Sensitivity), and it is prone to errors mainly due to contamination of the tip and/or rigid substrate, especially on biological samples (see supp. informations 3 and 4). The proposed standardized nanomechanical AFM procedure (SNAP) eliminates those errors by calculating the correct deflection sensitivity from the thermal fluctuations of the free cantilever with a known accurately and independently determined value of the spring constant (supp. information 5).

To prove the efficiency of the procedure, we first performed experiments on soft polyacrylamide gels (with mechanical properties comparable to those of cells but less intrinsic variability) and later on living cells, specifically confluent layers of MDCK-C11 cells. Experiments were performed with the same type of cantilever but with local AFM instruments (11 in total from 4 different manufacturers).

Errors in elastic modulus estimation due to uncertain cantilever calibration and instrumental errors^23^, especially determining force constant^24–27^ and its consequences in force spectroscopy measurements have been elucidated in previous work by others^28^. However, to our best knowledge, this is the first study aiming to address the consistency and reproducibility in mechanical measurements with AFM by measuring living cells in different labs using a variety of different AFM instruments.

## Results and Discussion

We propose a new standardized procedure that improves accuracy, or more exactly speaking consistency, and reproducibility of mechanical measurements by AFM. This improvement reduces significantly the technically derived variability of elastic moduli measured on soft samples and living cells. The procedure was tested in eleven different European labs proving its reliability independently of users and instruments. We neglect here other systematic errors, which are not instrumental. E.g. cells show viscoelastic behaviour where viscosity may be visible in force curves depending on scan rate. To accurately measure viscoelastic properties of samples other techniques have been derived, which will also benefit from the procedure described here, when they are based on AFM. Another point is limitations of the Hertz model used here for analysing indentation data. The Hertz model requires a linear response of an isotropic and homogenous sample. Strictly speaking all assumptions are not valid for cells, nevertheless in most applications of AFM the Hertz model is used. Sometimes this point is discussed in publications, e.g. by calling the derived elastic moduli as “apparent” Young’s moduli. Again, this systematic, hence not instrumental error, needs being considered; however it is beyond the scope of this paper. Mechanical measurements by AFM will benefit by using the procedure described here, since measurements will be reproducible and consistent independent from labs involved or instruments used.

**Figure 1:**
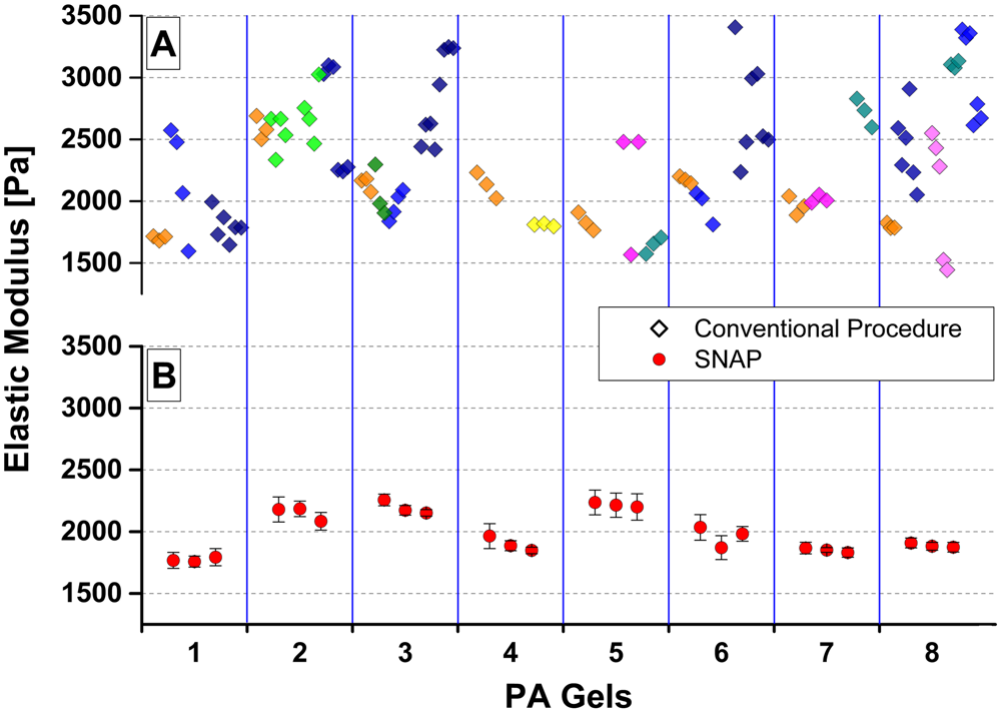
Comparison of mechanical properties of polyacrylamide gels measured with conventional procedure and with SNAP. (**A**) Elastic moduli of eight gels prepared centrally in Bremen and measured from the different participating labs. Each diamond marker indicates the mean value of the elastic modulus extracted from each force map; data were acquired and analysed using the conventional AFM procedure. Different marker colours stand for different AFMs from several manufacturers: JPK (NanoWizard III), Bruker (Catalyst, Nanoscope and Multimode), Park System (XE120) and Asylum (MFP3D). (**B**) Same eight polyacrylamide gels were measured in Bremen and elastic moduli were evaluated applying the standardized nanomechanical AFM procedure (SNAP). Each circle marker indicates the mean value of the elastic moduli extracted from a force map (made of 100 force curves). On each gel three force maps were recorded. Bars represent standard deviations.

Soft polyacrylamide gels were prepared in a single processing step from the same stock solution in Bremen and their elastic moduli were measured with colloidal probes using a MFP3D AFM. These gels were then sent to the participating labs and measured with the local AFMs. Afterwards each lab sent the measured sample to one of the other labs. In this way gels circulated all over Europe to get many data from different groups. All the results were collected and compared in Bremen. It turned out that values of elastic moduli were very different among each lab (Fig. 1A). This prompted us to search for potential error sources causing this inhomogeneity.

Why elastic moduli of gels were so different when measured in different labs? Several hypotheses that could contribute to this discrepancy were proposed:

- Hypothesis 1. The force constants used in analysis (calculated from the AFM thermal spectra) were wrong;
- Hypothesis 2. The different gels exhibited different elastic moduli (E) since their properties changed because of time or travel conditions;
- Hypothesis 3. The deflection sensitivity had been calibrated erroneously, which would affect the force and indentation values calculated (supp. info 2).

Other possible systematic (and hence constant) error sources like problems in calibrating AFM thermal spectra (supp. info 2) or in analysing thermals (supp. info 3) and the accuracy of the fitting procedure (supp. info 7) are discussed in the supplementary information.

Hypotheses 1 and 2 were quickly confuted. For hypothesis 1, we analysed our data in two ways: using the force constant from the thermal spectrum or the vibrometer determined value reported from the manufacturer. First approach corresponds to the normal way AFM experiments are done whereas the other relied on accurate knowledge of the force constant. However, there was no real improvement in the second approach: the variations in elastic moduli of different gels did not decrease. Thus, we could conclude that the consistency of the results did not depend on the instruments or the software used for analysis.

Hypothesis 2 was tested in another set of experiments where gels were measured again in Bremen with the same instrument and tip, the same deflection sensitivity and spring constant by one single user and under the same experimental and instrumental conditions (deflection sensitivity and spring constant) after circulation in several labs. Hence, these data demonstrated mechanical homogeneity and stability even after 6 months (Figure S8 in supp. material 6).

If we assume that the only (or major) error in the force constant determined by AFM thermal comes from an erroneous deflection sensitivity (Hypothesis 3), we could calculate a correction factor λ for the deflection signal, knowing a reliably calibrated force constant. We propose here an alternative procedure to the conventional calibration protocol: we suggest using an accurate value for the force constant, e.g. determined by a vibrometer, and calculate a correction factor λ for the deflection signal, based on the ratio of the force constant determined by an AFM thermal spectrum and the real value (see supporting information 5 for details). This approach is related to a method introduced some years ago to calibrate the force constant and deflection sensitivity by using an AFM thermal spectrum combined with a method to estimate the force constant using the Sader method^29^. However, the Sader method relies on determining the Q factor and a hydrodynamic correction factor, which is unique and has to be determined experimentally for each cantilever geometry^30–32^. Both quantities are difficult, i.e. error prone, to determine for low resonance frequency, soft cantilevers in liquids, as has been used here. Thus, we relied here on the spring constant as determined by a vibrometer. An alternative variation of the Sader method for calibration of cantilever spring constants has been recently proposed which overcomes these issues and might represent an alternative to the use of a vibrometer^33^. However, we propose using accurate force constants determined by interferometry, since this direct method promises better accuracy for soft cantilevers in liquid.

**Figure 2:**
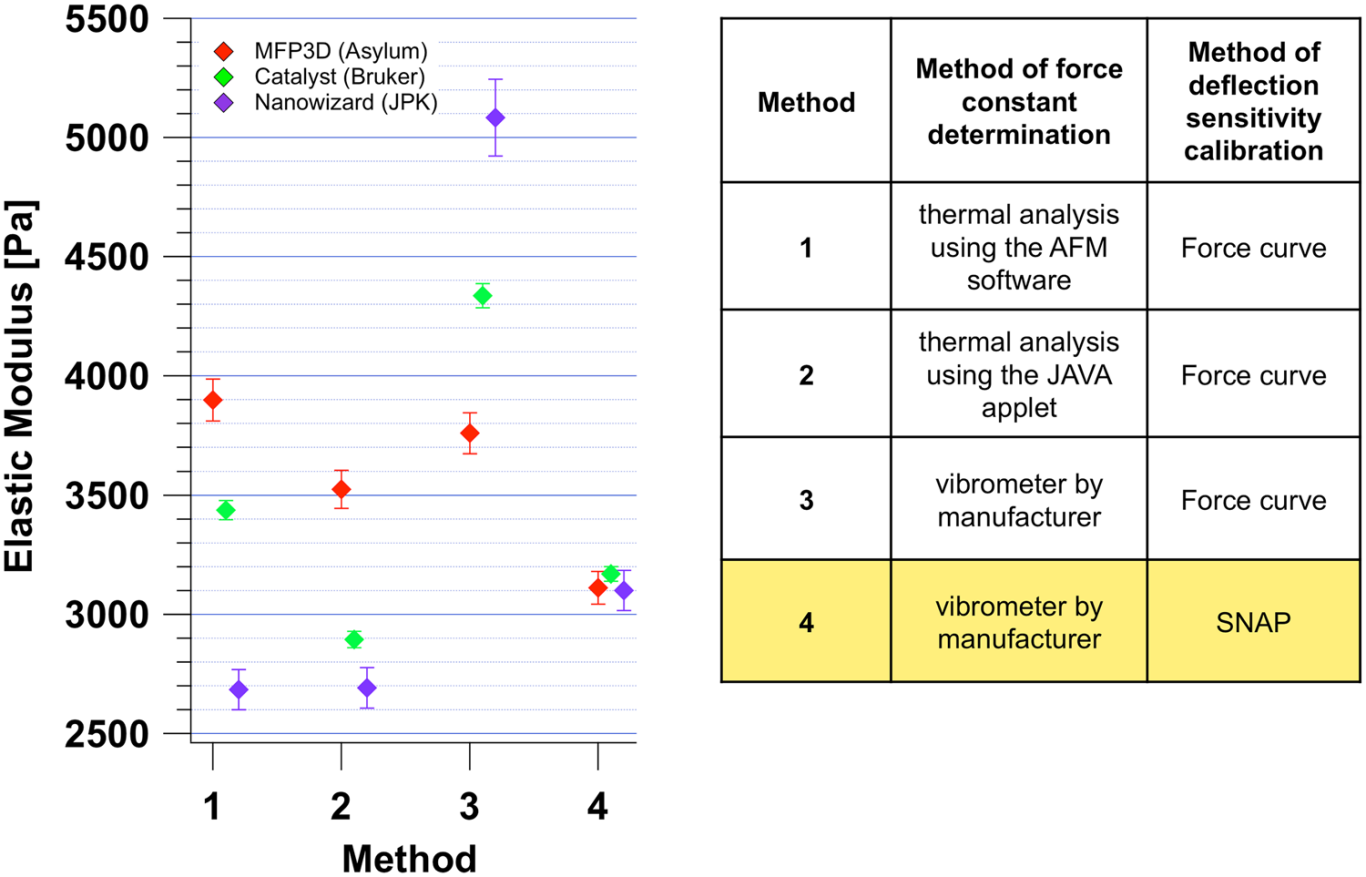
SNAP reduces the variability of mechanical measurements on the same gel in different locations. Elastic moduli of the same gel measured with three different instruments using the same kind of colloidal probes. The data were then analysed with different methods numbered from 1 to 4. In 1 to 3 ones, the deflection sensitivity was calibrated with a force curve on a stiff substrate and the force constant was determined from the analysis of the thermal with the AFM software (1); from the analysis of the thermal with the JAVA applet (2) and from the vibrometer measurements (3). In method 4, SNAP was applied: the force constant was measured using the vibrometer and deflection sensitivity was re-calibrated with the correction factor λ.

Figure 1B shows data of elastic modulus obtained on the same 8 gels presented in Fig. 1A, but recorded and analysed following our procedure from one user in one location. These data prove that the proposed procedure strongly reduces the variability of the measurements. However, the aim of this work was to test the procedure among different users and instruments, therefore successively, one single gel was independently measured with three different instruments (from Asylum, Bruker and JPK) in different locations but with the same kind of colloidal probe. Figure 2 shows a comparison of elastic modulus obtained from force maps recorded and analysed following the herein proposed SNAP, as well as with the other previous conventional methods; in fact, AFM data were analysed with the Hertz model using each time a force constant extracted following a different approach. In particular, by using the deflection sensitivity from force curves on a rigid substrate and force constant values calculated from the AFM thermal or measured with the vibrometer, a large variation in elastic moduli can be seen. Variability was reduced to about 1% (3127 Pa +/− 35 Pa) when analysed according to SNAP (Fig. 2). Therefore, this procedure proved to handle uncertainties in setting the cantilever deflection signal adequately, leaving only other factors, like tip geometry, as possible sources of systematic errors (Supporting Information 7).

After proving the effectiveness of the proposed procedure on soft polymeric samples, we proceeded in testing its reliability on biological samples as well: MDCK-C11 cell samples were prepared in one lab (Münster), sent to all the participating groups (the experiment was repeated twice), and data were analysed locally and additionally centrally with the custom-made software package in Bremen. We analysed data both with and without employing our proposed procedure using the correction factor λ (for more details see supp. info. 5 and 8).

**Figure 3:**
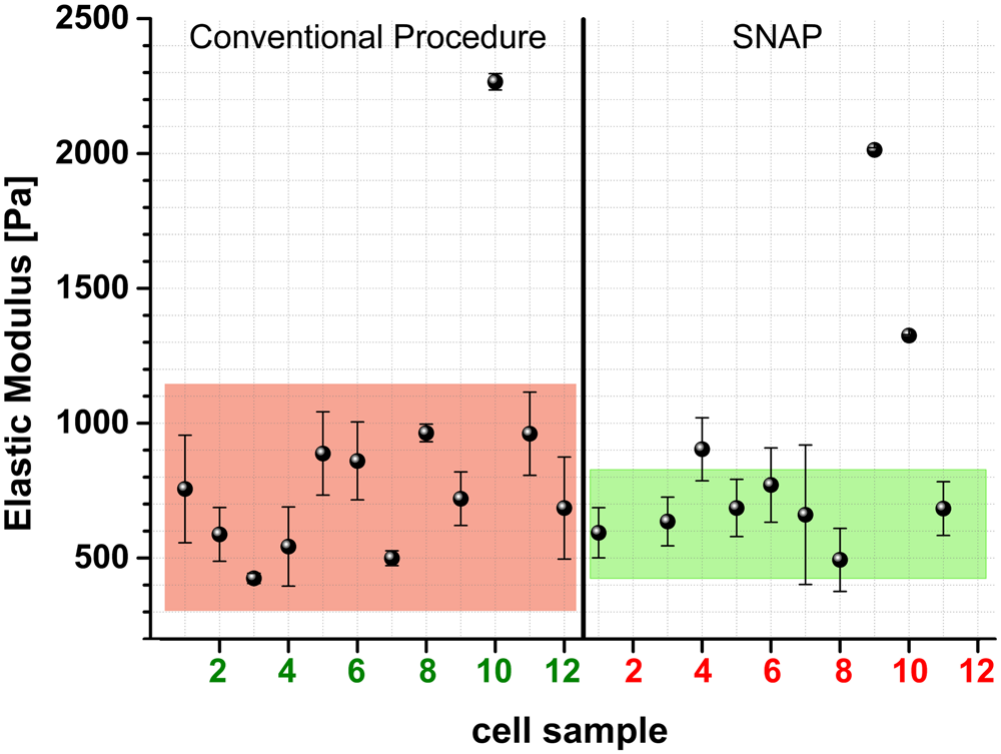
Mechanical properties of MDCK C11 cells. Elastic moduli were determined by participating labs with and without the application of SNAP (using the deflection sensitivity extracted from AFM thermal spectra with Java applet). Data represent peak value and the width of the histograms of Young’s moduli determined by fitting a Gaussian function locally around the peak value (see *Methods* section for details). The average and standard deviation of all typical values for each lab (i.e. each cell sample) are depicted as colour bars showing the increased reproducibility of SNAP (green) compared to the conventional (red) one.

SNAP leads to a substantial increase in consistency; in fact, implementing this correction the elastic modulus has been determined to be 655 +/− 171 Pa in comparison to 758 +/− 373 of the conventional procedure (Fig. 3). So, with the help of SNAP the consistency has been increased by a factor of 2.

**Figure 4:**
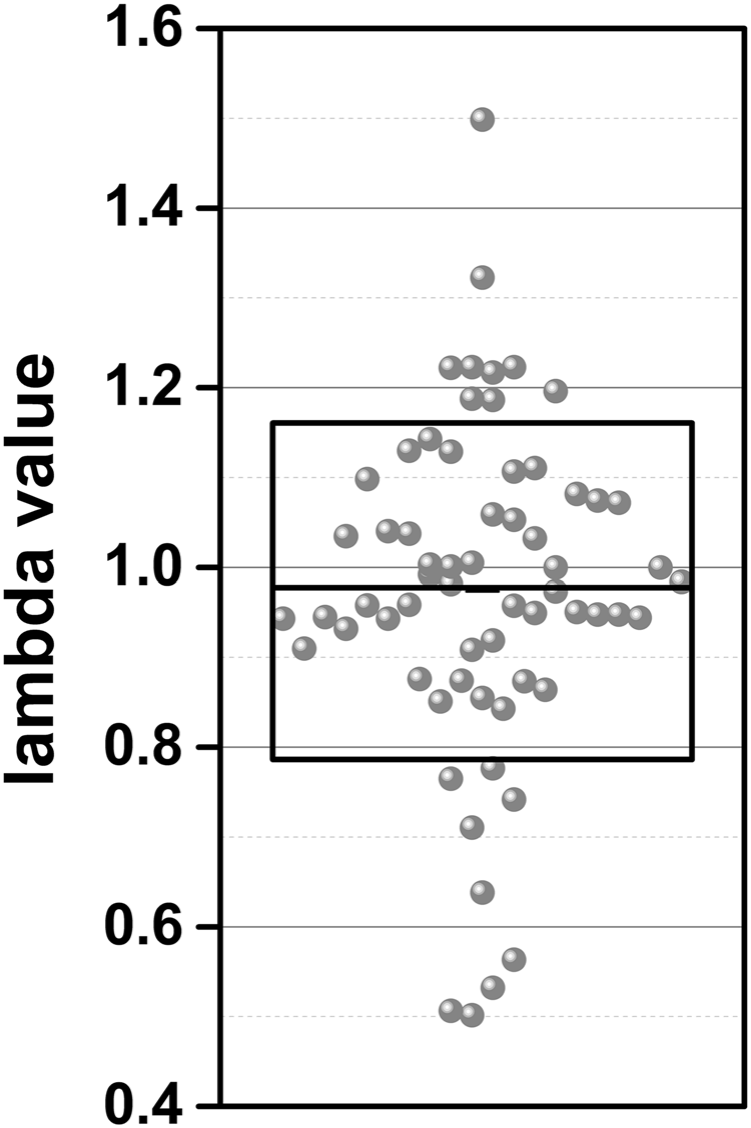
Values of the correction factor lambda (λ) identified for each applied SNAP. Lambda values presented as box-plot showing raw data (spheres), standard deviation (whiskers), 25 and 75 percentile (box) and the median (horizontal line).

Without SNAP the variation between experiments (labs) is very high. On average, i.e. averaging several independent measurements from different locations, we got similar values for the Young’s moduli. However, since the variation between experiments is very large, the confidence of an individual measurement is much poorer. The consistency and reproducibility of data obtained applying SNAP is two times higher than data obtained with the conventional procedure, hence demonstrating the benefit of the proposed procedure. The correction factor lambda (λ) was identified for each applied SNAP (Fig. 4). The lambda value indicates the relative deviation of those calculated from the conventionally obtained deflection sensitivity and denotes its relative error directly (λ 1.1 = 10% rel. error of deflection sensitivity). Figure 4 shows large variations of lambda with a mean of 0.97 and a standard deviation (SD) of 0.19 (n = 64). The data revealed that 98.4% (63) of all conventionally obtained values for the deflection sensitivity show errors, mostly in the range of 0–10% (34 out of 64) but nearly 50% in the range of 10–50% (30 out of 64). Obviously, determination of deflection sensitivity is a significant error source for any kind of AFM-based force measurements.

Biomechanics has been identified as a promising field that can contribute in the study of human diseases^34^ resulting, besides biological and functional changes, in alterations of physical and structural features of cells. A strong connection between diseases and cell mechanics has been found in several studies^35–37^.

Among all the available methods to study mechanical properties of cells, AFM is emerging as a powerful and versatile technique in the biomechanical field due to its ability to operate both as a tool for high-resolution topographical imaging and as a force sensor with pico-newton resolution^38, 39^. One of the biggest advantages of the AFM is its ability to perform measurements on biological samples under physiological conditions, without any further treatment.

Since its invention^40^ the number of publications involved in the use of AFM is continuously increasing. However, elastic moduli reported in the literature often show a large variability between different cell types, or between different labs even for the same cell type^15^. Beside natural occurring heterogeneity of biological samples, this is potentially caused by differences in protocols used for acquiring and analysing data and/or instrumental errors.

In this work, we investigated potential reasons for these variations and we developed a new procedure for data acquisition and analysis that has shown large improvement in consistency and reproducibility: so -called SNAP. Eleven research groups all over Europe have studied the properties of soft gels and living cells, proving the real efficiency of the proposed method.

By following the herein introduced procedure, we demonstrate that reproducibility, consistency, and the inter-laboratory comparability of biomechanical data can be improved considerably. The two main error sources in mechanical measurements of soft samples by AFM have been defined, namely, force constant and deflection sensitivity values. They are connected, since force constants are often determined by AFM thermals, which are based on the deflection sensitivity determination. In consequence, the elastic moduli derived from force curves exhibit large variations. In our studies, both sources of errors are handled adequately by SNAP, which relies on the accurate knowledge of the force constants of the cantilever, and uses this value to set the deflection sensitivity. After applying the proposed procedure, the errors present in gel measurements decreased from 30% down to 1% while in cell measurements the consistency increased by a factor of 2 (Figs 2 and 3).

Obviously, biologically derived variations of elasticity measurement results could not be reduced due to the nature of living cells but technical variations could be minimized by using this standardized procedure.

## Methods

### General materials

Acrylamide and bisacrylamide solutions were purchased from Bio-Rad. *N*,*N*,*N*′,*N*′-Tetramethylethylenediamine (TEMED), *N*-[3-(Trimethoxysilyl)propyl]ethylene- diamine silane and dichlorodimethylsilane solution were purchased from Sigma. Sodium hydroxide (NaOH) and ammonium persulphate (APS) were purchased from Merck. Glutaraldehyde, ethanol and other solvents were purchased from Panreac AppliChem. Rectangular cover glasses were provided by Thermo Scientific. MEM (E15-888), L-Glutamine, fetal bovine serum and penicillin/streptomycin were purchased from PAA Laboratories (Pasching, Austria).

### Polyacrylamide (PA) gel preparation

The PA solution was prepared by mixing 40% acrylamide and 2% bisacrylamide in ultrapure water (MilliQ systems, Molsheim, France). Finally, APS and TEMED were added to activate the polymerization. PA solution was poured between two glasses (microscope slide and glass coverslip) to obtain a smooth surface and to avoid the presence of oxygen that would inhibit the polymerization. The microscopy slides were treated with 0.1 M NaOH for 3 min, then *N*-[3-(Trimethoxysilyl)propyl]ethylenediamine silane solution was added for additional 3 min. After washing with ultrapure water three times for 10 min, each sample was covered with 0.5% glutaraldehyde for 30 min, then extensively washed again and finally dried. The upper glass coverslips were treated with dichlorodimethylsilane solution to create a hydrophobic surface reducing gel adhesion, hence facilitating the glass coverslip removal after gel polymerization, which lasted typically 30 minutes. Slides with gels were shipped in 50 mL polypropylene conical tubes submersed in ultrapure water. The diameter of the tube (30 mm) and the size of the slides (75 × 25 mm²) prevented contact between gels and the tube wall.

### AFM data acquisition

The basic idea of measuring mechanical properties of soft samples like cells measures sample indentation (δ) as a function of loading force (F) using a well-defined tip. In AFM, these quantities are not measured directly, but need to be calculated from the deflection of the cantilever (d) and the sample height (z) recorded as so-called force curves. The force can be calculated from the deflection (if the force constant is known) whereas the difference between height and deflection delivers indentation. To extract the elastic modulus from the force indentation data, we used the Hertz model for colloidal probes^41^ (for a review on applying the Hertz model to soft samples like cells, see ref. 39). This model is only applicable (in a strict sense) if certain conditions are complied with (like homogeneity and isotropy of the sample) and if the tip geometry is simple enough and well defined (which prompted us to choose spherical colloidal probes instead of the conventional pyramidal tips, see Supporting Information 7). However, even for complex samples like cells, the model is generally accepted and it is usually applied for data analysis.

### AFM experiments on PA gels

Preliminary experiments were performed on PA gels, with elasticity values in a comparable range to those of cells. Initially, gels were prepared and measured in Bremen, then sent to the other locations and circulating from lab to lab. Data were recorded with the respective local AFM using colloidal probes: spherical SiO_2_ beads with a diameter of 6.62 µm attached to a tipless PNP-TR-TL cantilever (nominal k = 0.08 N/m; CP-PNP-SiO-C-5 NanoAndMore, Karlsruhe, Germany). Force constants were measured for each cantilever by the manufacturer with a vibrometer and found to be around 50 mN/m. The vibrometer used was a Polytec (Waldbronn, Germany) OFV-5000 system with a DD-600 digital displacement decoder on a Mitutoyo (Japan) upright microscope with a 20x objective. A careful calibration of the deflection signal has been done on a stiff substrate (glass slide). Several force curves were recorded to achieve a reasonable average for the deflection sensitivity. Force volume maps were recorded with a scan rate of 1 Hz, travel range of 4 µm, tip velocity of 8 µm/s and trigger deflection of 50 nm (corresponding to a trigger force of 2.5 nN) in water at room temperature. The data were recorded in the centre of the gel; typically, 100 force curves were collected over an area of 10 × 10 µm². For a second round of experiments, in order to test the proposed procedure, one same gel was investigated by using three different AFMs (Asylum, Bruker and JPK). Several techniques to analyse data and to calibrate deflection sensitivity and force constant were compared with SNAP.

### Cell culture

The Madin–Darby canine kidney cell subclone C11 (MDCK strain II) resembles alpha-intercalated cells^42^ with a cubic to high-prismatic morphology. Cells were grown at 37 °C and maintained in modified minimum essential medium (MEM) containing Earl’s balanced salt solution supplemented with 2 mM L-Glutamine, 10% heat-inactivated fetal bovine serum (FCS), 50 IU/ml penicillin, and 50 µg/ml streptomycin in a 5% CO_2_-humidified incubator. Confluent cell layers were subcultured weekly by trypsinization. For AFM experiments, cells were seeded on the glass surface (18 mm well) of PTFE coated microscope slides (Diagnostic slides (IFA), product No. 61.100.24, Immuno-Cell, Mechelen, Belgium) and cultured for 5 days in the aforementioned medium^42^.

### Cell shipping

For shipping living cells to the participating labs, medium was exchanged against HEPES-Ringer buffer pH 7.4 (in mM: Hepes (N-(2-hydroxyethyl)piperazine-N′-2-ethane sulfonic acid) 10, NaCl 122.5, KCl 5.4, MgCl_2_ 0.8, CaCl_2_ 1.2, NaH_2_PO_4_ 1, and D-glucose 5.5). Slides with cells were shipped at room temperature in 50 mL conical polypropylene tubes filled with HEPES-Ringer buffer. The diameter of the tube (30 mm) and the size of the slides (75 × 25 mm²) prevented contact between cells and the tube wall. Insulated polystyrene boxes were used for express shipping and cells received their destination within 24 h.

### AFM experiments on cells

Each participating lab measured mechanical properties of MDCK-C11 cells with the local AFM, including Resolve, Multimode and Catalyst (Bruker, Santa Barbara, CA, USA), NanoWizard III (JPK, Berlin, Germany), MFP3D (Asylum Research, Santa Barbara, CA, USA) and XE120 (Park Systems, Suwon, S-Korea). Every lab received the same type of colloidal probe cantilever: tipless MLCT (Bruker, Santa Barbara CA USA) with a spherical polystyrene bead (diameter 10 µm) glued to the D-cantilever (nominal k = 0.03 N/m) (Novascan, Ames, IA, USA). The spring constant of each individual probe was measured by the cantilever manufacturer with a vibrometer, values ranged from 41 to 44 mN/m. Force curves were recorded with a scan rate of 1 Hz, travel range of 4 µm, tip velocity of 8 µm/s and trigger deflection of 50 nm (corresponding to a maximum force of 2.5 nN) in HEPES-Ringer buffer at room temperature. Since cells show viscoelastic behaviour it is of utmost importance to maintain the same settings for all data compared within this study. 100 curves were acquired over an area of 20 × 20 µm² in the form of force volumes. For each samples three force volumes were recorded at different areas, for a total of 300 curves for each experiment, hence for each laboratory. Data were analysed with and without applying SNAP to assess the actual benefits from the proposed method. The experimental steps have been standardized as much as possible: first, thermal tunes were recorded in the buffer afterwards a careful calibration of the deflection signal has been done on a stiff substrate (glass slide). A force curve was recorded to determine the deflection sensitivity. From this point, LASER position and photo detector were kept unchanged. Thermal tunes were analysed with the built-in software of each instrument and/or by custom protocols. Briefly, thermal fluctuation data of free cantilevers were recorded, the deflection signal was corrected for the differences in amplitude and deflection sensitivities (see supporting information 3), transformed in frequency space by the Fourier transform, and then a model function (single harmonic oscillator or Lorentzian) was fitted to obtain the total power, and hence the force constant (see supporting information 1). In addition, data were exported to be analysed in a custom-made Java applet, developed within this initiative. This applet is open source and publicly available^43^. We recommend here using a Lorentzian for thermals in liquids, since the fluctuations of a cantilever in water is highly over damped. In rare cases, we have seen that the thermal fluctuation of free cantilevers in air and liquids reproduce exactly the same force constant, as they should, however, in most cases rather large discrepancies (of up to 50%) between the two were detected. There may be some systematic instrumental errors (like instrument’s transfer function at low frequencies, which is optimized for stiff cantilevers in air, which can fail for thermals of soft cantilevers in liquid), but the main problem is due to errors in deflection sensitivity calibration (see supporting information 4, Figure S7).

### Standardized nanomechanical AFM procedure (SNAP)

Usually in AFM, the deflection sensitivity is determined from a force curve on a stiff substrate (which can be difficult in cell samples, since even bare regions will be covered with extracellular matrix components secreted by neighbouring cells). After deflection sensitivity is determined, the thermal fluctuations of the free cantilever are recorded, and by analysing the power spectral density (PSD) of these fluctuations the force constant is determined. If the deflection sensitivity is wrong (and our experience with cell samples shows that it will be wrong by 5 to 20%), the fluctuation data will be erroneously calibrated and the force constant will be wrong by roughly twice the error in the deflection signal. Thus, we propose an inverse procedure: determine the force constant by using some highly accurate method (like a vibrometer); record the thermal fluctuations and then adjust the deflection sensitivity such that the thermal results in the same exact force constant. This can either be done before the experiment, by applying a correction factor to the deflection sensitivity, or during offline analysis, if the software allows to re-calibrate the data. Most software packages provided by the AFM manufacturer do not allow re-calibrating data after acquiring, thus we developed software solutions using the data analysis package Igor (Wavemetrics, Lake Oswego, OR, USA). We also provide an open source JAVA applet that allows to re-calibrate and analyse single force curves. A step-by-step description of SNAP can be found in supporting information 5 and 8.

### Analysis of peak values of elastic moduli histograms

In each laboratory three force maps were recorded on samples (gels or cells) with 10 by 10 force curves over an area of 20 µm. From each force map, the histogram of the Young’s moduli was displayed and the peak was calculated. This was motivated by the fact that the histograms were asymmetric and this made the conventional measures (mean and median) not really appropriate, thus we calculated the peak value and a measure for the width of this peak. Since the peak value in a histogram depends strongly on bin size, we first determined the maximum in each histogram; then a fit of a Gaussian was applied in the vicinity of the peak; more precisely in a region defined by the half height of the maximum value. This allowed localizing the peak with higher precision than the bin size, and the results were practically independent of bin size. The width of the Gaussian is used as a measure of the width of the peak. If the distribution was Gaussian, then this number would be identical with the standard deviation of the data, however, here the distribution is not Gaussian. Nevertheless, since the fit is done only locally around the peak, this part of the histogram is reasonably symmetrical to justify the use of a Gaussian fit.

## Electronic supplementary material

Supporting Information
