# Supplementary material for: Standardized Nanomechanical Atomic Force Microscopy Procedure (SNAP) for Measuring Soft and Biological Samples
Source: Sci Rep. 2017 Jul 11;7:5117. doi: 10.1038/s41598-017-05383-0 (PMC5505948; doi:10.1038/s41598-017-05383-0)
Supplement: Supplementary file 1 — Supporting Information [file 41598_2017_5383_MOESM1_ESM.pdf]

SUPPORTING INFORMATION for

# Standardized Nanomechanical Atomic Force Microscopy Procedure (SNAP) for Measuring Soft and Biological Samples

Hermann Schillers[1], Carmela Rianna[2], Jens Schäpe[2], Tomas Luque[3], Holger Doschke[2], Mike Wälte[1], Juan José Uriarte[3], Noelia Campillo[3], Georgios PA Michanetzis[4], Justyna Bobrowska[5], Andra Dumitru[6], Elena T. Herruzo[6], Simone Bovio[7], Pierre Parot[8,9], Massimiliano Galluzzi[10,11,12], Alessandro Podestà[10], Luca Puricelli[10], Simon Scheuring[13,14,15], Yannis Missirlis[4], Ricardo Garcia[6], Michael Odorico[9,16], Jean-Marie Teulon[9,17], Frank Lafont[7], Malgorzata Lekka[5], Felix Rico[13], Annafrancesca Rigato[13], Jean-Luc Pellequer[9,17], Hans Oberleithner[1], Daniel Navajas[3], Manfred Radmacher[2*]

[1] Institute of Physiology II, University of Münster, 48149 Münster, Germany
[2] Institute of Biophysics, University of Bremen, 28359 Bremen, Germany
[3] Institute for Bioengineering of Catalonia, University of Barcelona, and CIBER Enfermedades Respiratorias, 08028 Barcelona, Spain
[4] Department of Mechanical Engineering & Aeronautics, University of Patras, 265 04 Patras, Greece
[5] Institute of Nuclear Physics, Polish Academy of Sciences, PL-31342 Krakow, Poland
[6] Instituto de Ciencia de Materiales de Madrid, CSIC, Sor Juana Ines de la Cruz 3. 28049 Madrid, Spain
[7] CMPI-CIIL, CNRS UMR 8204 - INSERM U1019, Institut Pasteur de Lille - Univ Lille, F-59019 Lille Cedex
[8] BIAM, CEA, Aix-Marseille Univ., Saint-Paul-Lez-Durance 13108, France
[9] CEA Marcoule, iBEB, Department of Biochemistry and Nuclear Toxicology, F-30207 Bagnols-sur-Cèze, France
[10] CIMaINa and Department of Physics, Università degli Studi di Milano, via Celoria 16, 20133 Milano, Italy
[11] College of Materials Science and Engineering, Shenzhen Key Laboratory of Polymer Science and Technology, Guangdong Research Center for Interfacial Engineering of Functional Materials, Nanshan District Key Lab for Biopolymers and Safety Evaluation, Shenzhen University, Shenzhen 518060, PR China
[12] College of Optoelectronic Engineering, Key Laboratory of Optoelectronic Devices and System of Ministry of Education and Guangdong Province, Shenzhen University, Shenzhen 518060, PR China
[13] U1006 INSERM, Aix-Marseille Université, Parc Scientifique et Technologique de Luminy, 13009 Marseille, France
[14] Department of Physiology and Biophysics, Weill Cornell Medicine, New York, NY 10065
[15] Department of Anesthesiology, Weill Cornell Medicine, New York, NY 10065
[16] ICSM, UMR 5257, CEA, CNRS, ENSCM, Univ. Montpellier, Site de Marcoule – Bât. 426, BP 17171, 30207 Bagnols-sur-Cèze, France
[17] Univ. Grenoble Alpes, CEA, CNRS, IBS, F-38000 Grenoble, France

**Supporting Information**

Supporting Information 1: AFM data analysis 3
- *Thermal data - determination of force constants* 3
- *Indentation data* 5
- *Analysis of force indentation data* 6

Supporting Information 2: Calibration of AFM deflection Sensitivity 9

Supporting Information 3: Deflection sensitivity of a vibrating cantilever 11
- *Eigenmodes of a vibrating or supported cantilever* 11
- *Amplitude sensitivity correction: the κ factor* 12
- *Effect of cantilever tilt* 13

Supporting Information 4: Thermal vibration spectra of cantilevers 16
- *Power spectral density of a single harmonic oscillator (SHO)* 17
- *Power spectral density of a Lorentzian oscillator* 18
- *PSDs of vibrating cantilevers in air and water* 19

Supporting Information 5:
SNAP: standardized nanomechanical AFM procedure 20

Supporting Information 6: Application of SNAP on soft gels 22

Supporting Information 7: Errors in determining elastic properties 23
- *Estimation of errors in determining the elastic modulus* 23
- *Tip geometry and probe radius* 24
- *Force Constants* 25
- *Deflection Signal* 27
- *Accuracy of the Hertz fitting procedure - contact point* 27
- *Error Propagation* 28

Supporting Information 8: The SNAP protocol sheet 30

Supporting Information 9: Typical Force Data on Gels 33

Supporting Information 10: Typical Force Data on Cells 36

Supporting Information References 39

## Supporting Information 1: AFM data analysis

*Thermal data - determination of force constants*

Several methods have been discussed in the literature for calibrating the force constant of AFM cantilevers[1,2]. Most prominent are the added-mass method[3], the use of reference cantilevers[4], and the thermal tune method[5]. An alternative is a calculation of the force constant from the geometrical dimensions[6,7]. The latter is not used here because it requires the knowledge of phenomenological factors and works best for stiffer cantilevers than those used here.

Because the added mass method is destructive and the reference cantilever method is cumbersome, most often the thermal noise method is used. The principle of the thermal noise method is very elegant; however, it needs to be discussed here in some detail, since there are a few pitfalls with this method. The physics behind it is the Boltzmann's equipartition theorem: each degree of freedom of any physical system (e.g. an AFM cantilever) will have a thermal energy of 1/2 $k_B$ T, where $k_B$ is the Boltzmann's constant and T is the absolute temperature. If k is the cantilever's spring constant and x is the vibration amplitude, this reads as follows:

$$\frac{1}{2}k\langle x^2\rangle = \frac{1}{2}k_B T \qquad \text{(S1)}$$

The angle brackets denote the time average of the amplitude of vibration. AFM cantilevers are not single harmonic oscillators, but exhibit several vibrational modes, each mode actually carries 1/2 $k_BT$ of energy. Because each mode has a distinct resonance frequency, it is easily possible to separate the modes from each other in the frequency domain. Thus, AFMs usually record the fluctuations of the AFM cantilever and present the data as its Fourier transform, i.e. as the power spectral density (PSD). In this sense eq. S1 is written here only for didactic reasons and should not be understood literally.

The vibrational modes of a cantilever are different for a free cantilever (as used in a thermal) and a supported cantilever (as in contact with the sample), so the sensitivity factor used for converting photodiode signal (measured in volts) to cantilever deflection (in nanometres) is different for the two situations. Since the amplitude sensitivity of a free cantilever cannot be calibrated easily in AFM, usually a correction factor κ is used, to calculate the amplitude signal from the measured deflection signal.

$$\kappa = \frac{DeflectionSensitivity_{free}}{DeflectionSensitivity_{fixed}} \qquad \text{(S2)}$$

$$x = \kappa * d \qquad \text{(S3)}$$

In our experiments the value for κ slightly deviates from the common values (1.08 or 1.09), for reasons given in the section "*Amplitude sensitivity correction: the κ factor*" in the Supporting information 3, amplitude sensitivity correction: the *k* factor.

Combining eq. S2 and S3 we can calculate the force constant, as soon as we know the time average of the deflection signal squared:

$$k = \frac{k_B T}{\kappa^2 \langle d^2 \rangle} = \frac{1}{\kappa^2} * \frac{k_B T}{\langle d^2 \rangle} = e * \frac{k_B T}{\langle d^2 \rangle} \quad \text{(S4)}$$

In some of the literature (e.g. [8]) a correction factor $e=1/\kappa^2$ can also be found. Depending on the instrument, the deflection signal is directly corrected by the κ factor as described above or the result of the power spectral density analysis is corrected accordingly.

The mean square displacement could either be calculated by averaging over the deflection signal as a function of time, or by integrating the PSD over a specified bandwidth. Alternatively, and often more accurate, a model function is fitted to the PSD and then the integration is done analytically over the model function to yield the mean square displacement. An obvious choice of a model function is a single harmonic oscillator, which will give the following result for the force constant (see supporting information 4):

$$k_{SHO} = \frac{2 k_B T}{\pi \omega_0 Q {A_{DC}}^2} \quad \text{(S5)}$$

where Q, $A_{DC}$, and $\omega_0$ are the quality factor, the amplitude at DC (at frequency 0) and the resonance frequency, respectively. The derivation is given in the supporting information 4, Power spectral density of a single harmonic oscillator (SHO).

From a physical point of view, the above derivation holds for cantilevers in air or when immersed in liquids. In liquids, like water, damping is increased, but more importantly the effective mass is also increased, since water is dragged along with the cantilever. This results in a decrease of resonance frequency and in a widening of the resonance peak[9], which aggravates recording of good thermal spectra in water. Since the soft cantilevers used here are highly damped in liquids (low Q), it has been suggested to use the Lorentzian model instead[8]. Pirzer and Hugel have extended this model such that the phenomenological parameters $C_1$ and $C_2$ can be understood in terms of physical quantities (see supporting information 4, eq. S35 and S36). Here we will stick with the nomenclature used in most instruments:

$$k_{Lorentz} = \frac{k_B T}{\frac{C_1}{\sqrt{C_2}} * \left[\frac{\pi}{2} + arctan\left(\frac{\omega_0}{\sqrt{C_2}}\right)\right]} \quad \text{(S6)}$$

Soft cantilevers as used here, qualify as highly damped systems, independently of whether the data are acquired in air or in liquids. In many cases, the results of the two models do not deviate much, however as a general rule the fit of a SHO followed air data better, whereas the Lorentzian model looked better for liquid data. The difference only becomes apparent at low

amplitudes, i.e. at the flanks of the peaks. Here a logarithmic display can help, which is not available in all built-in software as provided by the AFM manufacturers.

*Indentation data*

Indentation data on gels and cells were analysed within the instrument's software, or alternatively by custom routines including software written in the analysis package Igor (Wavemetrics, Oswego, WA). A fit of the Hertz/Sneddon model (assuming a given force constant and tip geometry) was applied to the data to obtain the Young's modulus (E, elastic modulus) values. A functionally identical version of the fit procedure is available as an open source Java online tool[10].

In a force curve we measure the deflection as a function of z height of the sample. Since the offset of the deflection is arbitrary, we need to subtract the offset $d_0$ (the deflection reading of the free cantilever) before calculating the force F:

$$F = k_c * (d - d_0) \tag{S7}$$

The deflection offset $d_0$ is usually determined by averaging some part of the force curve, where the tip is not in contact with the sample surface. This requires that the force curve be reasonably flat, i.e. long-range interaction forces are negligible and no other artefacts are present in the data. Occasionally we have found that force curves have a tilted baseline. This may be due to laser light being reflected from the sample, detector-cantilever-laser alignment issues, or mechanical drift in the optical detection system.

The indentation δ is the difference between deflection and z-height, which also needs to be calculated relative to the contact point ($z_0$) at which the tip contacts the sample:

$$\delta = (z - z_0) - (d - d_0) \tag{S8}$$

Mathematically, we could combine the two offsets in one, however for procedural reasons it is easier to keep them separated. Once we have calculated force and indentation, we can employ an appropriate model to calculate the elastic properties of the sample, namely the elastic modulus. The simplest model is the Hertz model[11] and its various implementations, e.g. originally derived by Sneddon[12]. Strictly speaking, the assumptions of the Hertz model (purely elastic sample of infinite thickness, homogeneous and isotropic sample, no adhesion) are all not given for cells, it works very well in many cases and is therefore widely used. The advantage of the Hertz model is that it allows comparing measurements between different groups, since it takes different tip geometries into account. Since cells behave in a viscoelastic manner, which means that energy is dissipated into the cell when they are indented by the AFM tip (hysteresis in the force-deformation curve) we had to keep the viscous part (e.g. hysteresis) constant and small by using a probe velocity of 8 μm/s

*Analysis of force indentation data*

In AFM measurements, the Hertz model relations for force versus indentation data are usually used for the following three tip geometries: paraboloid, cone, and pyramid. The paraboloid model is also applied when using spherical probes. This is valid for small indentations only, i.e. much smaller than the radius, which is often neglected. Since the contact problem of a rigid pyramidal tip indenting an elastic half-space is not solvable analytically, most often the cone model is used instead in AFM studies. Alternatively, approximations could be used for pyramidal tip data, as outlined by Rico et al[14], based on the derivations from Bilodeau[15] and Barber et al[16]. We will use here the relation for a four-sided pyramid. Unfortunately, the pyramid model is not implemented in commercial AFM software packages. We have used here custom-built analysis software based on the package Igor, where all tip models are available. Details of the analysis procedure have been described elsewhere[14,17].

$$F_{paraboloid} = \frac{4}{3} * \frac{E}{1-\nu^2} * \sqrt{R} * \delta^{3/2} \quad \text{(S9)}$$

$$F_{cone} = \frac{2}{\pi} * \frac{E}{1-\nu^2} * tan(\alpha) * \delta^2 \quad \text{(S10)}$$

$$F_{pyramid} = \frac{1}{\sqrt{2}} * \frac{E}{1-\nu^2} * tan(\alpha) * \delta^2 \quad \text{(S11)}$$

where R is the radius of curvature at the apex of the paraboloid (equivalent to the radius of spherical probes, when this model is applied to that case), $\alpha$ is the half-opening angle of the cone or a four-sided pyramid, respectively, and $\nu$ is the Poisson ratio. However, some of the recent tip geometries commercially available are not regular, presenting different angles for each face. A reasonable approximation for that case would be using the average of all angles $\theta_i$, or, better, the average of all tan($\theta_i$), which is the geometry factor in the force-indentation relationship.

The most critical point of the analysis procedure is the determination of the contact point between the probing tip and the sample surface. On a stiff sample a force curve will exhibit a sharp transition at the contact point, where the slope jumps from 0 (free cantilever) to 1 (in contact). In soft samples, the Hertz model actually predicts a smooth transition, where not only the deflection data itself, but also the slope will smoothly increase from zero (see figure S1).

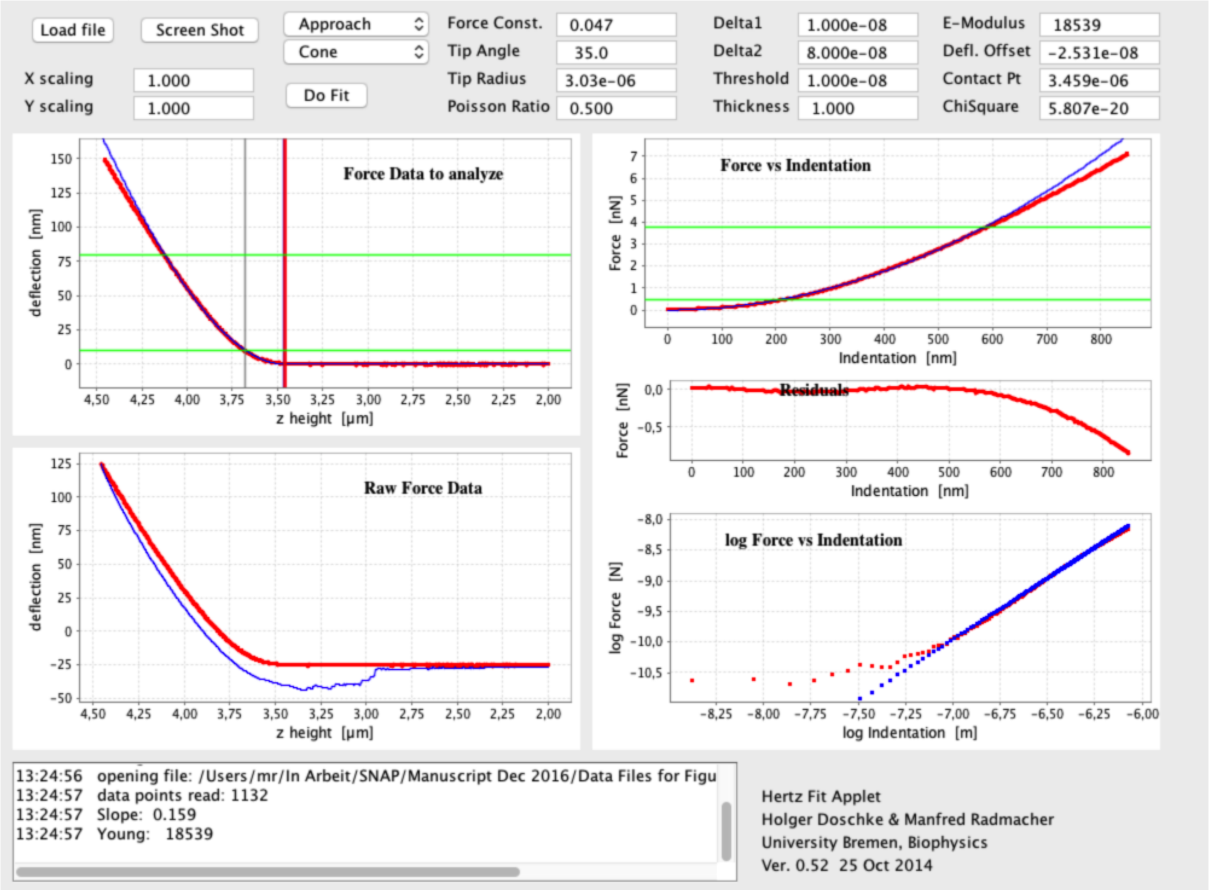


**Figure S1**

Analysis of a typical force curve recorded with a colloidal tip on a soft polyacrylamide gel. The lower left panel shows the measured raw deflection and z-height data (the red curve is showing AFM data and the blue curve the Hertz fit). After subtracting the offsets of deflection and z-height (zero force and contact point) the data to be further analysed are plotted in the top left panel. From these data the indentation and deflection are calculated using eq. S7 and S8, which are plotted on a linear or logarithmic scale on the right. A fit of the appropriate Hertz model (sphere here) results in the elastic modulus. From this value a theoretical force vs. indentation trace (blue: simulated force) and a theoretical force curve is calculated, which follows very nicely the measured data (red trace). Deviations are visible in the log-log display of force vs. indentation (lower right panel) for very small forces (below 20 pN) due to the thermal noise of the cantilever.

Since the soft cantilevers we are using here will have a thermal force noise level on the order of 20 pN, simple procedures, like thresholding, will result in contact point values, which are systematically off. We have used the following procedure here to determine the contact point:

(1) Determining the deflection offset by averaging some portion of the force curve data (typically the first 10 % of the data).
(2) Introducing a first guess of the contact point position as the z height where the deflection is larger than some threshold value (typically 10 nm). This value will be systematically wrong!
(3) Calculating the force vs. indentation data based on known offsets values (from points 1 and 2). A fit of the Hertz model to the data within some range, safely above the threshold value (typically between 50 nm and 200 nm deflection), is done. Here, $z_0$ is included as a fit parameter, so in addition to the elastic modulus E a new estimate of the contact point is obtained

(4) This new contact point position is used to recalculate force vs. indentation data and a Hertz fit is done again.
(5) We repeat points 3 and 4 until the contact point does not change considerably any more (typically 3 fit rounds are sufficient).

The final result of this procedure is shown in figure S1, where the four values for the contact point (one after thresholding, three by the successive rounds of fit) are depicted as vertical lines in the upper left panel. The grey vertical line shows the contact point determined by thresholding, which is systematically different from the other three obtained by the fit, which change only marginally, showing that our procedure converges nicely.

To test how well this fitting procedure works, we have analysed artificial force indentation data with added noise (see supporting information 7). We have picked a noise value of 0.5 nm in the deflection signal, which corresponds to the thermal noise level typical in our AFM data. In a second simulation, a noise level of 2 nm has been picked, which could serve as an upper limit of noise in AFMs.

Noise in the data will lead to a misjudgement of elastic modulus, but also in the contact point. The latter is actually more important, since if the contact point is wrong by same value, this will translate in different indentation values and thus affect the elastic modulus calculated. We have calculated, for different analysis ranges, the elastic moduli of our fake data with a nominal modulus of 10 kPa. When including only a small portion of the data in our fit, errors in elastic moduli can be very substantial, i.e. up to 5 % for the data with 0.5 nm and up to 20 % for the noisy data (see supporting information 7, figure S11). If the analysis range is extended to more than 300 nm and the contact point is included in the analysis, errors will become very small: less than 0.2 % in the 0.5 nm noise data, and less than 1 % in the noisy data (2 nm noise). There is a clear correlation between misjudgement of contact point and calculated elastic modulus. This supports the above argumentation that the most critical part of data analysis is the way the contact point is determined. In our experience, it is necessary to control the range of data included in the fit and to include the contact point as a parameter adjusted by the fit itself. Only an iterative procedure, i.e. applying the fit routine several times to the data, and adjusting the contact point and recalculating the force and indentation data accordingly, will lead to reasonable elastic values, if the results of this iteration process are converging. The importance of determination of the contact point in analysing mechanical data, has been pointed out previously[18,19].

## Supporting Information 2: Calibration of AFM deflection sensitivity

Figure S2 shows a typical force curve recorded with a pyramidal tip on a clean glass substrate in air and in water, respectively. When using soft cantilevers, we see large adhesion forces (capillary forces) in air, resulting in non-linearities in the deflection signal due to saturation and/or friction. This may result in differences in slope between approach and retract curves. Therefore, we do recommend using only force curves in liquids (water, buffer, cell culture medium) to set the deflection sensitivity.

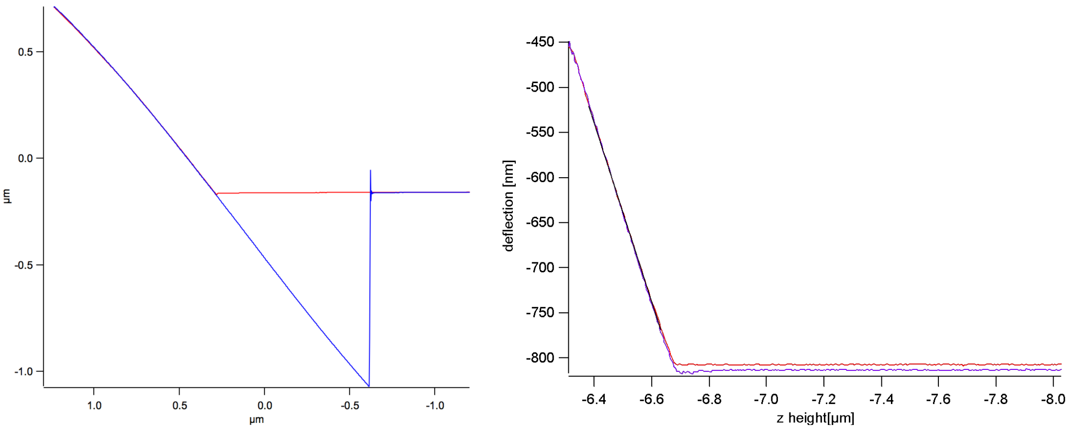


**Figure S2**
Force curve taken in air (left) and water (right) with a soft cantilever (nominal force constant 10 mN/m). Very large adhesion can be seen on retract. This results in high friction and saturation of the cantilever sensor in contact, which makes it difficult to determine the slope accurately. The force curve taken in water shows virtually no adhesion and no difference between approach and retract curve is visible in the contact region.

Even in perfect conditions, i.e. in water with negligible adhesion, there are still some non-linearities present in the data. This is apparent when looking at force curves with sufficient detail. Figure S3 shows two force curves, recorded in water on a clean glass support, with a pyramidal tip and a colloidal probe as used in this study. When zooming in the region around the contact point very little electrostatic repulsion can be seen in the case of the pyramidal probe (on the order of 1 nm corresponding to 10 pN), but some substantial rounding of the deflection in the case of the colloidal probe is visible. This could possibly be due to a thin (20-40 nm) layer of contaminants on these tips, which is inevitable after using them repeatedly.

When calculating the slope of the force curve for different parts of this force curve, we get values, which differ slightly from each other. Figure S4 shows the mean slope calculating from sliding windows of 50 nm width along the force curve. The ones centred around 25 nm give much smaller values than the ones at larger deflections. However, even in the range between 100 to 350 nm, where the data looks reasonably linear to the eye, the variations are on the order of 7 % for the pyramidal probe and 10 % for the colloidal probe.
Since these deviations from the slope 1 are probably due to systematic errors (non-linearities in the deflections sensor, or in piezo, or due to friction and interaction forces between tip and sample), the situation does not improve by averaging several force curves as is often done.

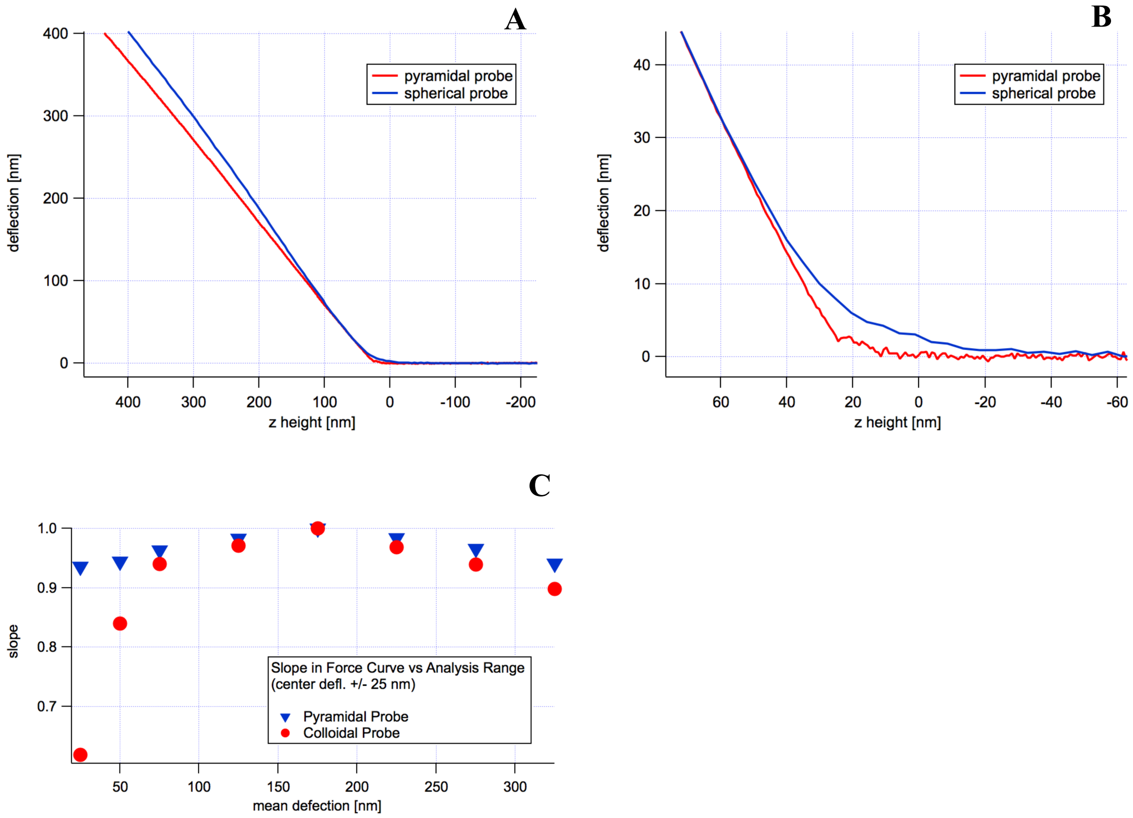


**Figure S3**

Two force curves taken in water with a pyramidal (red trace in A & B) and a colloidal probe (6.62 µm silicon dioxide bead) (blue trace in A & B). Panel B is a zoom-in of the entire dataset (A), showing that close to the contact point we get deviations from a perfect force curve. In the case of the pyramidal probe this may be due to electrostatic repulsion, in the case of the colloidal probe this may reflect the fact that the surface of the bead this may be due to some surface contamination layer. In C the slope values calculated in sliding windows of 50 nm width calculated from the force data of A are shown. Even when disregarding the extreme values at 25 nm close to the contact point, the variation in slope is 7 % for the pyramidal probe and 10 % for the colloidal probe.

Figure S4 shows a histogram of slopes of 100 force curves measured over an area of 10 µm times 10 µm. The variation between force curves, when analysed in the same way is very small, hence the systematic errors discussed in figure S3 is larger than the measurement error reflected in repeated measurements.

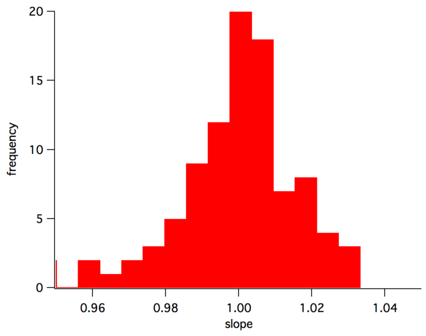


**Figure S4**

Histogram of the slope of 100 force curves taken over an area of 10 by 10 µm. The standard deviation is about 0.01.

## Supporting Information 3: Deflection sensitivity of a vibrating cantilever

*Eigenmodes of a vibrating or supported cantilever*

For a rectangular beam the normal modes will have the following shapes[20]:

$$x_{(y)} = A * \left[\left(sin(\alpha_i) + sinh(\alpha_i)\right) * \left(cos\left(\alpha_i * \frac{y}{L}\right) - cosh\left(\alpha_i * \frac{y}{L}\right)\right) - -\left(cos(\alpha_i) + cosh(\alpha_i)\right) * \left(sin\left(\alpha_i * \frac{y}{L}\right) - sinh\left(\alpha_i * \frac{y}{L}\right)\right)\right] \quad \text{(S12)}$$

where x is the amplitude at position y of a cantilever of length L. The parameters $\alpha_i$ depend on the boundary conditions, thus being different for free and fixed beams, corresponding to the cases of a free cantilever and a cantilever in contact with a support, respectively.

The resonance angular frequency $\omega_i$ of each mode is given by:

$$\omega_i = \alpha_i^2 * \frac{Eh^2}{12\rho L^2}$$

(S13)

(S13)

| | Free cantilever | | Fixed cantilever | |
|---|---|---|---|---|
| Mode | $\alpha_i$ | $\omega_i$ [kHz] | $\alpha_i$ | $\omega_i$ [kHz] |
| 0 | 1.8751 | $\omega_0$ | 3.9266 | 4.39 * $\omega_0$ |
| 1 | 4.69409 | 6.27 * $\omega_0$ | 7.06858 | 14.21 * $\omega_0$ |
| 2 | 7.85476 | 17.54 * $\omega_0$ | 10.2102 | 29.65 * $\omega_0$ |
| 3 | 10.9955 | 34.39 * $\omega_0$ | 13.3518 | 50.70 * $\omega_0$ |

**Table S1**
Values for the first four $\alpha_i$ for a free cantilever and a cantilever in contact with the sample, and the resulting fundamental frequencies.

The modes are well separated in frequency, thus the analysis of thermal fluctuations of a cantilever in frequency space will easily separate the different modes. Figure S5 shows the first three modes of a free and a fixed AFM cantilever.

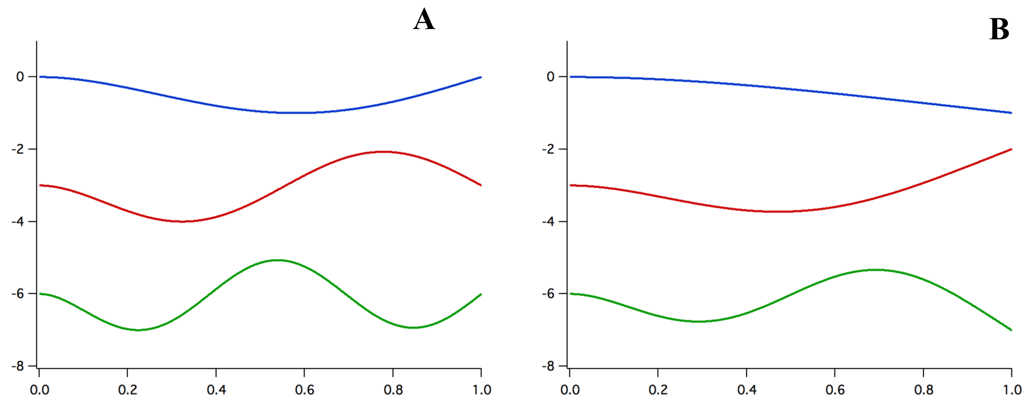


**Figure S5**
First three normal modes of a freely oscillating (A) and fixed (B) AFM cantilever. As can be seen all modes will change the orientation at the end of the cantilever, thus they will be picked up (to some degree) by the optical lever detection. The easiest way to discriminate between different modes is by frequency, i.e. looking at the PSD (power spectral density) of the deflection signal.

*Amplitude sensitivity correction: the κ factor*

As shown before, the mode of vibration of a free and a supported cantilever is different, and hence the orientation at its end will be different for the two cases, even for the same deflection, or better amplitude in the case of the free cantilever. Thus, the signal measured by the optical lever scheme, and calibrated for an end supported cantilever (a tip in contact with a rigid substrate in a force curve) needs to be corrected to give the right amplitude value.
This correction factor $\kappa$ can be derived from first principles, taking into account the different eigenmodes of oscillations in the case of a rectangular cantilever.

Based on the work by Butt and Jaschke, Proksch et al suggested a value of 1.09[22] for κ.

In a subsequent work Hutter[23] modified this correction considering cantilever tilt and finite tip height:

$$\kappa = 1.09 * \frac{1-\frac{2D}{L}tan(\propto)}{1-\frac{3D}{2L}tan(\alpha)} \qquad \text{(S14)}$$

Here D is the tip height, L the cantilever length and α the tilt angle of the cantilever. For pyramidal tips typical values (D = 4μm, L = 320 μm, α = 12 degrees) we will get the following value, which is used in this study:

$$\kappa = 1.09 * 0.9989 = 1.088 \sim 1.09 \qquad \text{(S15)}$$

Hutter[23] suggested a further correction, which considers cantilever tilt by adding a cos(α) term to eq. S14. However, as outlined (but not supported by a derivation) by Ben Ohler[24] this correction is not necessary (see below: effect of cantilever tilt).

The applicability of this κ factor has been tested experimentally by directly measuring the deflection of free and fixed end cantilevers with a vibrometer[25]. However, the above result is only exact for rectangular cantilevers and not for triangular cantilevers as used in this study and in most other AFM studies. There is a number of publications, where κ values have been determined by finite element simulations (e.g. see Pirzer and Hugel[8]), or more elaborate models, like equivalent point mass models[26], which basically come to similar values. The best guess, which is accepted by the community, for the soft triangular cantilevers used in this study seems to be a value of 1.10, which is used throughout this manuscript. *Nota bene*: in most commercial instruments a deflection correction factor of 1.08 or 1.09 is set by default by the manufacturer, which needs to be corrected.

*Effect of cantilever tilt*

Hutter[23] pointed out that an error is made in calibration of the force constant by a thermal tune, when neglecting the tilt angle of the cantilever. In short: in AFM the deflection perpendicular to the surface is measured, or more accurately the deflection sensitivity is set by a force curve, thus it is calibrated such that the deflection signal measures only the component of the true motion of the cantilever. This effect is in addition to the above-discussed point of considering the different modes of motion of a free and a supported cantilever, which are dealt with the κ factor.
So, to obtain the true force constant of the cantilever, we need to multiply the amplitude of motion by a factor of 1 / cos(α), thus the force constant obtained from the thermal tune using eq. S33 or S39 needs to be multiplied by a factor 1 / $\cos^2(\alpha)$ to obtain the true force constant.
However, as it has been pointed out by Ohler[24], in AFM we do not use the true force constant of an AFM cantilever, but only its component, which is perpendicular to the surface. Again here the tilt angle has to be considered. Naively, one would expect that this could be taken care of by an additional correction factor cos(α). Ben Ohler stated, without derivation, that actually a correction factor of $\cos^2(\alpha)$ is appropriate here. In consequence, the two factors cancel and can be neglected, which is done in all instruments and in virtually all literature on this topic.
Since, the derivation has not been published yet, we will give the argument here:

Let's assume we have calibrated a cantilever using the thermal tune in an AFM with a tilt angle α and obtained a value $k_{AFM}$. Since we have measured only a component of the cantilever's true motion, we need to correct the deflection signal by a factor of 1 / cos(α), or we get the true force constant $k_{true}$ by the following expression:

$$k_{true} = cos^2(\alpha) * k_{AFM} \quad \text{S16)}$$

This can be understood by the following rationale. The measured deflection signal d in AFM is actually identical to the z-motion of the sample Δz. The true deflection of the cantilever Δx can be written as:

$$d = \Delta z \ = \Delta x \ * \ cos\alpha \quad \text{(S17)}$$

Here α is the tilt angle of the cantilever (see figure S6).

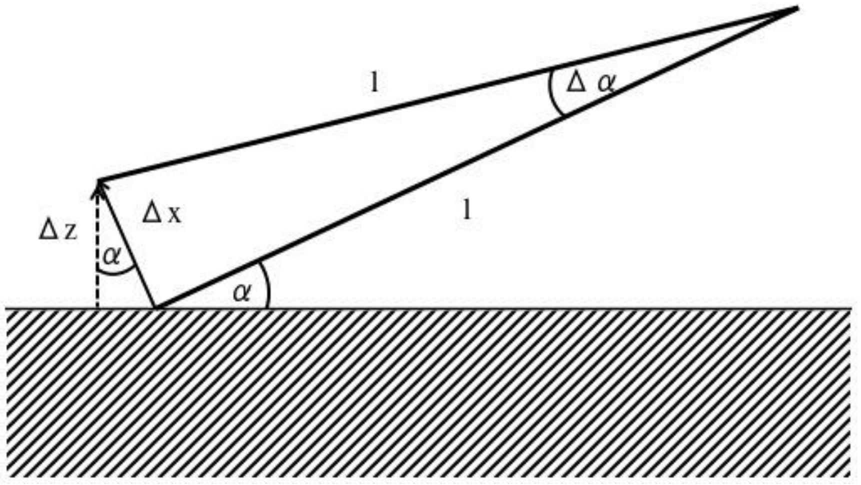


**Figure S6**
Schematic drawing illustrating cantilever tilt and the parameters used in the text

Consequently, we need to distinguish between the apparent force measured in AFM $F_{AFM}$ and the true force acting perpendicularly on the cantilever $F_{true}$. The work to deflect the cantilever can then be expressed as true force times true deflection, or apparent force times apparent deflection, which needs to be identical since we describe the same process here in two different ways:

$$W_{AFM} = F_{AFM} * \Delta z \quad \text{(S18a)}$$
$$W_{true} = F_{true} * \Delta x \quad \text{(S18b)}$$

The forces can be expressed by their respective force constant and the corresponding distance:

$$F_{AFM} * \Delta z = F_{true} * \Delta x \quad \text{(S19a)}$$
$$k_{AFM} * \Delta z^2 = k_{true} * \Delta x^2 \quad \text{(S19b)}$$

By rewriting eq. S19b and using eq. S17, we get:

$$k_{AFM} = k_{true} * \frac{\Delta x^2}{\Delta x^2 * cos^2\alpha} \quad \text{(S20)}$$

$$k_{AFM} = k_{true} * \frac{1}{cos^2\alpha} \quad \text{(S21)}$$

Eq. S21 is identical with S16.

Using an AFM thermal for determining the force constant, we do not need to consider cantilever tilt. If we just use the apparent deflection signal, we will actually only measure a component of

the true deflection, hence we will get the apparent force constant and not the true constant, which is actually what we need in AFM measurements.

However, if the force constant is measured in a vibrometer, which will measure the true deflection, we will get the true force constant. This has to be corrected for cantilever tilt angle, when the cantilever is used in AFM.

## Supporting Information 4: Thermal vibration spectra of cantilevers

The thermal vibration spectrum of a cantilever can be understood by looking at a dampened single harmonic oscillator (SHO). This will reproduce the spectrum for one vibration mode, or in other words the higher modes will need to be modelled by another SHO, which may have a different force constant and resonance frequency. A SHO is characterized by its resonance frequency, force constant, mass, and damping coefficient. The resonance frequency and the force constant are measurable quantities, whereas mass and damping will be effective quantities, which are hard to derive from first principles due to the complicated motion of a cantilever in a viscous medium (air or water). Since damping for the soft cantilevers is high, even in air, it has been suggested to use a Lorentzian spectrum as an alternative. We have used both and found that a SHO fits best our data in air, whereas a Lorentzian seems to work better in water (see below).

A vibrating cantilever will obey Boltzmann's equipartition theorem: in each vibrational mode the average kinetic and potential energy will be 1/2 $k_B$ T each, where $k_B$ is Boltzmann's constant and T is the absolute temperature. The average potential energy of the vibrating cantilever is given by multiplying its force constant k with the time average of the amplitude squared $<x^2>$, often referred to as mean square displacement MSD:

$$\frac{1}{2}k\langle x^2\rangle = \frac{1}{2}k_B T \quad \text{(S22)}$$

To calculate the MSD in real space, we need first to subtract the average value $x_0$ from the displacement.

$$x_0 = \lim_{T\to\infty}\frac{1}{T}\int_0^T x\,dt \quad \text{(S23)}$$

The mean square displacement will then be:

$$\langle x^2\rangle = \lim_{T\to\infty}\frac{1}{T}\int_0^T (x - x_0)^{\,2} dt \quad \text{(S24)}$$

If the mean square displacement has been measured directly, or it has been calculated from the Power Spectral Density, we can calculate the stiffness:

$$k = \frac{k_B T}{\langle x^2\rangle} \quad \text{(S25)}$$

Since it is difficult (actually impossible) to disentangle the contributions of the different oscillating modes in the time domain, we need to process our data in the frequency domain. This approach is feasible since the eigenmodes are well separated in frequency space.

*Power spectral density of a single harmonic oscillator (SHO)*

The SHO with an external (thermal) force, which has a white power spectrum, will obey the following equation of motion:

$$m\ddot{x} + m\gamma\dot{x} + kx = F_{Thermal} \qquad \text{(S26)}$$

If driven at the frequency ω the resulting amplitude will be:

$$A_\omega = \frac{F_0}{m}\sqrt{\frac{1}{(\omega_0{}^2-\omega^2)^2+\omega^2\gamma^2}} \qquad \text{(S27)}$$

This can be rewritten in:

$$A_\omega = A_{DC}\omega_0{}^2\frac{1}{\sqrt{(\omega_0{}^2-\omega^2)^2+\frac{\omega_0{}^2\omega^2}{Q^2}}} \qquad \text{(S28)}$$

or in terms of power:

$$P_\omega = A_{DC}^2 * \omega_0{}^4\frac{1}{(\omega_0{}^2-\omega^2)^2+\frac{\omega_0{}^2\omega^2}{Q^2}} \qquad \text{(S29)}$$

The thermal force is assumed to be white noise; so all frequencies are excited with the same amount of force. The total energy in the oscillator is given by Boltzmann's equipartition theorem. Since Parseval's theorem connects frequency space with real space, we can find the following relations:

If we have measured data $x_t$ in the time domain, the Fourier transform $x_\omega$ will be given by:

$$x_\omega = \frac{1}{\sqrt{2\pi}}\int_{-\infty}^{\infty} x_t e^{-i\omega t}\, dt \qquad \text{(S30)}$$

Parseval's theorem says:

$$\langle x^2\rangle = \int_{-\infty}^{\infty} x_t{}^2 dt = \int_{-\infty}^{\infty} x_\omega{}^2 d\omega \qquad \text{(S31)}$$

We can use this result in combination with equation (S25) to find an expression for the force constant:

$$k = \frac{k_B T}{\langle x^2\rangle} = \frac{k_B T}{\int_{-\infty}^{\infty} x_\omega{}^2 d\omega} = \frac{k_B T}{\int_{-\infty}^{\infty} A_\omega{}^2 d\omega} \qquad \text{(S32)}$$

$$k = \frac{2k_B T}{\pi\omega_0 Q A_{DC}{}^2} \qquad \text{(S33)}$$

*Power spectral density of a Lorentzian oscillator*

Since the soft cantilevers used here are highly dampened (in air the Q is on the order of 10, in water it may even be on the order of 1) Pirzer and Hugel [8,27] have suggested to use a Lorentzian PSD instead of the PSD of a SHO (eq. S29):

$$P_\omega^{Lorentz} = \frac{C_1}{(\omega-\omega_0)^2+C_2} + A_{White}^2 \quad \text{(S34)}$$

Here C1 and C2 are phenomenological parameters, which can be related to physical quantities, by the following substitutions:

$$C_1 = \frac{A_{DC}^2 * {\omega_0}^2}{4Q^2} \quad \text{(S35)}$$

$$C_2 = \frac{{\omega_0}^2}{4Q^2} = \frac{C_1}{A_{DC}^2} \quad \text{(S36)}$$

$$P_\omega^{Lorentz} = \frac{A_{DC}^2 * {\omega_0}^2}{4Q^2} * \frac{1}{(\omega-\omega_0)^2+\frac{{\omega_0}^2}{4Q^2}} + A_{White}^2 \quad \text{(S37)}$$

Integrating equation (S34 or S37) will result in the following mean square displacement[1]:

$$\langle x^2 \rangle = \frac{C_1}{\sqrt{C_2}} * \left[\frac{\pi}{2} + arctan\left(\frac{\omega_0}{\sqrt{C_2}}\right)\right] \quad \text{(S38)}$$

$$\langle x^2 \rangle = \frac{A_{DC}^2 * \omega_0}{2Q} * \left[\frac{\pi}{2} + arctan(2Q)\right] \quad \text{(S39)}$$

For low damping, i.e. in air, Q will be large and the square brackets can be approximated by a value of π. Here, we use the exact formula (eq. S38, S39), since we want to apply it to high damping, i.e. low Q cases.

Combining eq. S38 and eq. S39 we get the final result:

$$k_{Lorentz} = \frac{k_B T}{\frac{C_1}{\sqrt{C_2}} * \left[\frac{\pi}{2} + arctan\left(\frac{\omega_0}{\sqrt{C_2}}\right)\right]} \quad \text{(S40)}$$

The alternative form using the replacements (eq. S35 and S36) will look like:

$$k_{Lorentz} = \frac{k_B T}{\frac{A_{DC}^2 * \omega_0}{2Q} * \left[\frac{\pi}{2} + arctan(2Q)\right]} \quad \text{(S41)}$$

[1] The analytical integration has been done with Wolfram Mathematica Online Integrator: http://integrals.wolfram.com/index.jsp

*PSDs of vibrating cantilevers in air and water*

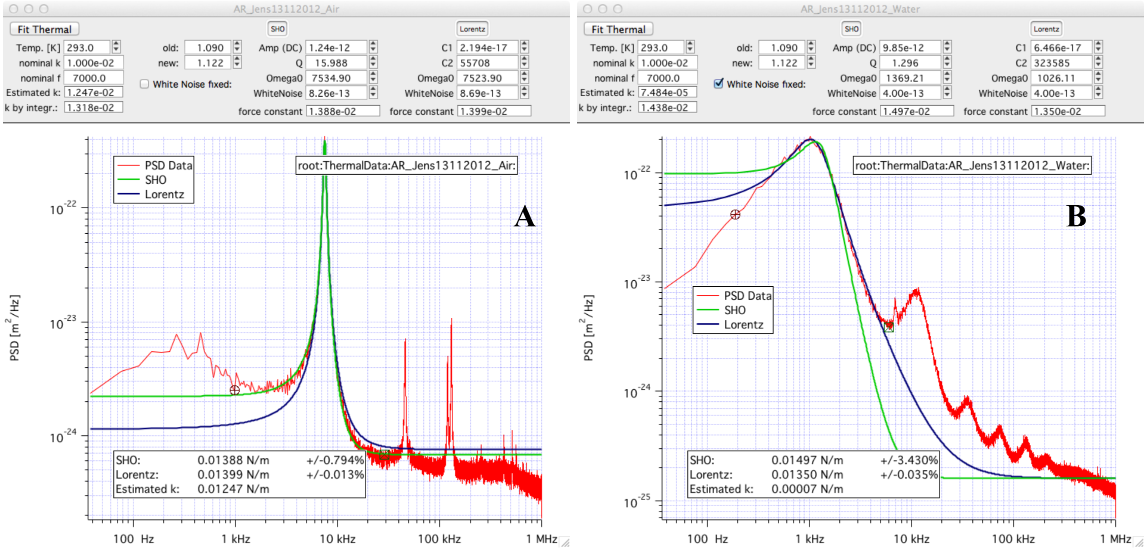


**Figure S7**
PSD of thermal noise of the same soft cantilever recorded in air (A) and in water (B). Data are acquired with a MFP3D instrument and analysed in Igor. Both data are fitted by a Lorentzian and a SHO model to calculate the force constant. Values agree reasonably well for all cases and are on the order of 14 mN/m.

Besides numerical errors due to fitting the model function, there are only two main error sources in this procedure:

(1) Frequency dependent noise in the instrument will show up as additional PSD, thus artificially resulting in a force constant, which is lower than the true value. In AFM usually noise filter (low pass filters) are implemented that will reduce high-frequency noise. In state of the art instruments this filter will have a cut-off frequency of 1 MHz or even higher. So they are not relevant for the soft cantilevers used here. Low frequency noise, which may be due to thermal drift in the instrument or environmental noise like building vibrations, is a more serious problem, especially for the soft cantilevers used here in liquids, where the resonance drops to 1 kHz. In some instruments a high pass filter is implemented, to cut off low-frequency noise. This will result in substantial errors in thermals.

(2) The force constant will in both cases be proportional to the inverse of the deflection signal squared. So, if the deflection signal has not been calibrated correctly, the force constant will be wrong as well. This is a serious source of errors, because errors in the deflection will enter quadratically the force constant.

## Supporting Information 5: The SNAP: standardized nanomechanical AFM procedure

For highly accurate and reproducible mechanical measurements of soft and biological samples we found the following step-by-step protocol to give the best results:

1. Use a cantilever with well-characterized force constant (e.g. using a vibrometer or a reference cantilever to measure the force constant).
2. Use a tip with well-defined geometry. Currently this asks for spherical probes. In the future, it is conceivable that pyramidal tips are sufficiently defined as well (cylindrical tips may become an interesting alternative[28] once they become commercially available).
3. Calibrate the deflection signal in the same buffer that will be used later in the real experiment (e.g. water or cell buffer).
4. Do not change laser adjustment or instrument settings, which will affect the deflection calibration.
5. Record a thermal tune.
6. Analyse the thermal tune to obtain the apparent force constant in the AFM ($k_{AFM}$) and adjust the cantilever deflection calculating the correction factor λ as defined later (this could be done with the help of our open source Java applet).
7. Record force curves or best force maps, which will allow you to correlate position and elastic moduli. This is of particular interest for non-homogeneous samples like cells, where the elastic properties strongly vary over the cell area (central or peripheral region).
8. Analyse the force curves with the Hertz model or any other appropriate model to extract elastic moduli values.

For step 6, if we have a very accurate value for the force constant, e.g. by vibrometer, $k_{IF}$ and a second value determined by an AFM thermal $k_{AFM}$ (which shall be wrong because the deflection signal has been calibrated erroneously) we can calculate a correction factor λ, which will give the correct deflection d* when being multiplied with the measured deflection d:

$$d^* = \lambda * d \qquad \text{(S42)}$$

Since the force constant k is proportional to the PSD (see supporting information 4), we can write the following proportionalities. $k_{AFM}$ will be proportional to the measured deflection signal squared. Since d is erroneous, $k_{AFM}$ will be as well.

$$k_{AFM} \propto \frac{1}{d^2} \qquad \text{(S43)}$$

If d* is the accurately calibrated deflection signal, it will be proportional to the accurate force constant $k_{IF}$.

$$k_{IF} \propto \frac{1}{d^{*2}} = \frac{1}{(\lambda * d)^2} \qquad \text{(S44)}$$

However $k_{IF}$ should be first corrected with the tilt angle of the cantilever α (see supporting information 3) under which it is mounted in the AFM.

$$k_{IFcorrected} = k_{IF} \cdot \frac{1}{\cos^2 \theta} \qquad \text{(S45)}$$

So, we can calculate the correction factor λ from the ratio of the force constants, determined by AFM thermal and vibrometer:

$$\lambda = \sqrt{\frac{k_{AFM}}{k_{IFcorrected}}} \qquad \text{(S46)}$$

Once we know the correction factor, we can calculate d* from eq. S42, write it in the software and do not change anymore the experimental settings.

## Supporting Information 6: Application of SNAP on soft gels

Initially SNAP was tested on polyacrylamide gels, with elasticity values comparable to those of living cells. Gels were prepared in Bremen and mechanical properties were measured with the local AFM before sending samples to the other participating labs. When measuring gel elasticity with the same instrument, elastic moduli were very homogeneous. However, when measurements were performed from different users in different locations, values were very different (see Figure 1 of the main text). To test the stability of the gels after many months circulating in different labs, in another set of experiments mechanical properties of gels were measured again in Bremen with the same instrument and tip, the same deflection sensitivity and spring constant by one single user and under the same experimental and instrumental conditions (deflection sensitivity and spring constant) after circulation in several labs. Data showed that their properties were stable after six months from the initial preparation (Figure S8)

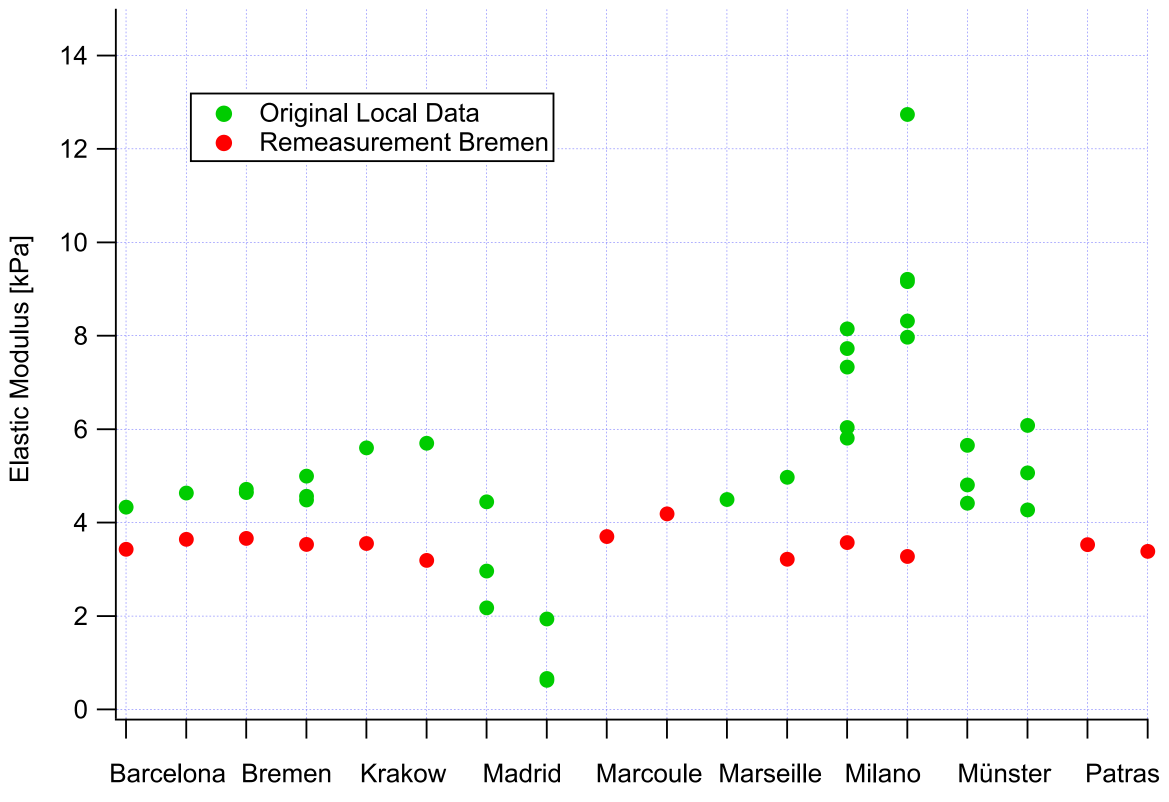


**Figure S8**
Elastic moduli of nine polyacrylamide gels prepared centrally in Bremen measured from the different participating labs. Each marker indicates the mean value of the elastic modulus extracted from each force map (three force maps per gel). Red dots show elastic moduli of the same polyacrylamide gels re-measured in Bremen after cycling through all participating labs, with the same instrument and tip by one single user and under the same experimental and instrumental conditions.

## Supporting Information 7: Errors in determining elastic properties

*Estimation of errors in determining the elastic modulus*

In order to discuss possible error sources, we want to rewrite equations S9 and S11 (the Hertz model for a spherical and pyramidal indenter, respectively) in terms of the measurable quantities deflection d and height z:

$$F_{paraboloid} = k_c * (d - d_0) = \frac{4}{3} * \frac{E}{1-\nu^2} * \sqrt{R} * (z - z_0 - d + d_0)^{3/2} \qquad \text{(S47)}$$

$$F_{pyramid} = k_c * (d - d_0) = \frac{1}{\sqrt{2}} * \frac{E}{1-\nu^2} * tan(\alpha) * (z - z_0 - d + d_0)^2 \qquad \text{(S48)}$$

The offset $d_0$ can be determined very accurately (<1 %) assuming that force curves are reasonably horizontal in the part of data, where the tip is not in contact with the sample. As discussed above determining the contact point $z_0$ is from a mathematical and a practical point of view very difficult. With our data and data processing routines the error in the elastic modulus E resulting from this point can be estimated to be below 1 % (typically 0.5 %).

Since in most commercial instruments, the z height is either measured by a sensor built in the piezo, in some instruments force curves are run in closed loop, so that creep and non-linearity are ruled out to a large extent. It is very important that in the force curve deflection data is plotted as a function of true z height (either the z position sensor signal or the z height signal applied in the case of closed loop scanners) and not as a function of z-voltage applied to the piezo, as has been done in the "old days" of AFM. If we assume that state of the art position sensors are used, which often have an accuracy of better than 1 nm, we can neglect errors in the z signal as well.

The Poisson ratio is usually assumed to be 0.5 for cells, since the cytoskeleton of cells behaves like a soft gel, where the volume is conserved during compression. The same argument applies to the gels used here. To avoid a discussion here, we could resort to determining the reduced E-modulus $\frac{E}{1-\nu^2}$ instead of the E-modulus E itself, but for the sake of simplicity of the argument we will follow here the common procedure to assume a value of 0.5 for the Poisson ratio.

So, we are left with three final and major error sources: errors in the force constant of the cantilever, in the tip geometry (either radius R for colloidal probes, or opening angle for pyramidal tips), and in the deflection signal.

*Tip geometry and probe radius*

Sharp pyramidal tips present some issues when used for nanomechanical characterization of soft samples, like living cells (see Ref. [30] for a detailed discussion). Shortly, sharp tips, with their radius of curvature typically below 50nm, cause the application of high stress, with large induced strain, in soft samples, also at small applied forces. Indentation is typically larger than the tip radius. The basic assumptions of the most used contact mechanical models, including the Hertz model, are typically not respected. Moreover, significant deviations from the nominal geometries are frequent with sharp tips, the evaluation of the relevant geometrical parameters (the side angles, the tip radius) being not straightforward. We have characterized by Scanning Electron Microscopy the tip angle values of sharp pyramidal tips and found discrepancies from the nominal values as large as 20%; these errors will then directly translate in an error of 10-20% in the Young's modulus, since the tip angle will enter at least linearity (by the tangent) in E values (Eq. S48).
We decided to use micrometer-sized, spherical probes (colloidal probes), since they represent a better alternative to sharp tips in terms of well-defined (and well characterizable) geometry and best fitting to contact mechanical models [30].

When using large spherical indenters of radius R, in the fitting procedure aimed at extracting the Young’s modulus value, the latter is proportional to $1/\sqrt{R}$ (Eq. S49). It is important either characterizing accurately the value of this parameter, or relying on a certification provided by the colloidal probe manufacturer. Since the error on the tip radius propagates via the square root, a target accuracy of 1% on the determination of the probe radius would be enough, as the resulting error in the elastic modulus will be smaller. Such 1% accuracy in the determination of the probe radius is typically obtained when the colloidal probe is characterized by means of reverse imaging against a spiked grating (like the TGT1 by NT-MDT)[29]. We have characterized some of the $SiO_2$ colloidal probes following the procedure described in detail in Refs [29-30] and found that the diameter deviates by less than 50 nm from the nominal value (6.62 μm).

While the radius of colloidal probes is typically reasonably well characterized and conserved within a batch, it is recommended to monitor the probe status since surface contamination (during the gluing of the sphere or the mechanical analysis), as well as intrinsic morphological defects of the micro-spheres used to assemble the probes, are not unlikely.
In Figure S9 we provide some examples of such defects, whose impact on the consistency of the mechanical measurement can be important, though not easily predictable. All images represent inverted AFM images of colloidal probes, obtained by scanning the probes across a TGT1 spiked grating. Each spike provides an independent replica of the probe shape, including surface defects and contamination. In order to highlight the residual roughness of the probe, in some cases the underlying spherical baseline has been subtracted. It should be noted that in general the surface defects proved to be firmly attached to the sphere surface, so to resist to prolonged scrubbing against the sharp needles of the grating at high applied forces. Some defects are as tall as a few hundred nanometres. If not properly removed (i.e. by cleaning the

probe in suitable detergents and solvents, like ethanol, Alconox, or Helyzime), such defects can significantly modify the local contact geometry, impacting on the accuracy of the mechanical analysis.

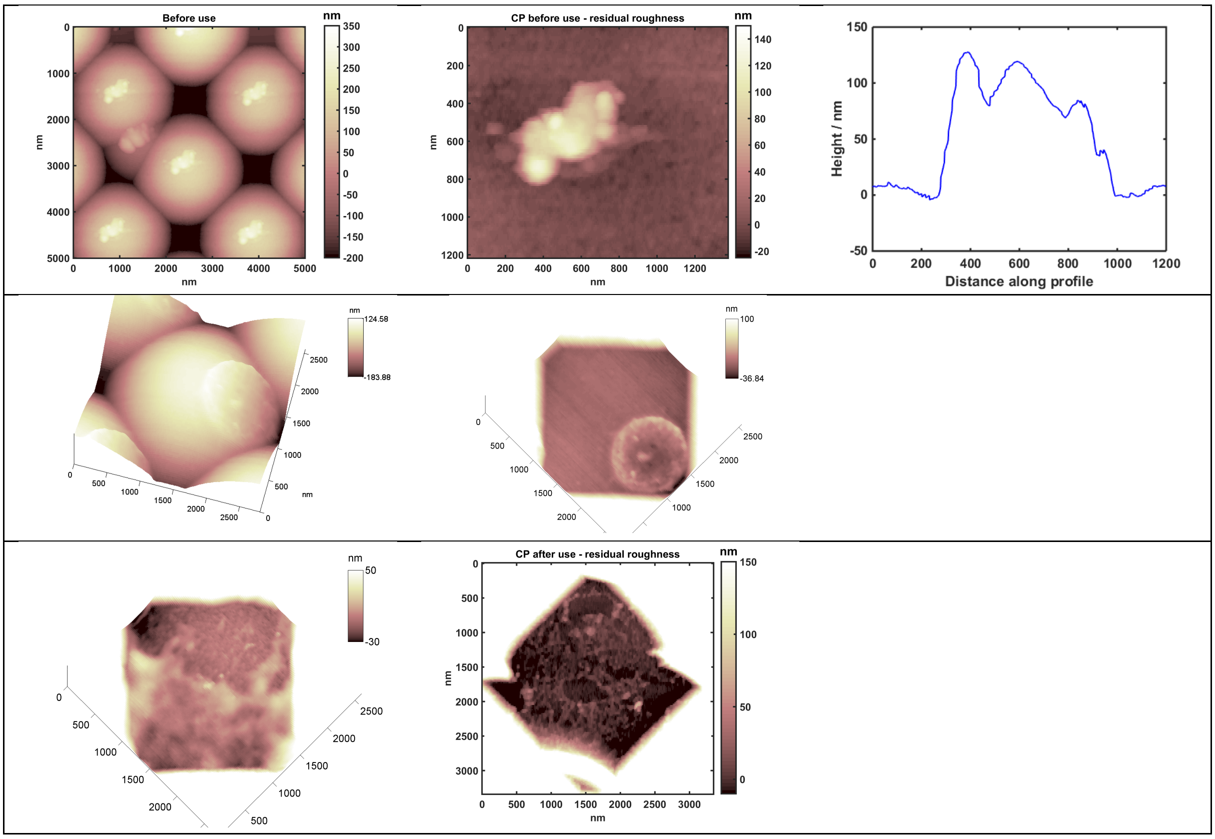


**Figure S9**
Surface contamination and defects observed by AFM reverse imaging on colloidal probes, after, and sometimes before, use. First row: a defect present on the probe surface since the beginning of the analysis. Due to its size, it clearly bring the effective contact geometry far from the sphere on flat regime. Second row: another original surface defect, probably a remnant of the glue used to attach the sphere to the tipless cantilever. Third rows: after use on soft samples (cells and gels), the surface of the probe gets easily coated by a layer of material, whose thickness can be of a few tens of nanometers. A similar contamination should not sensibly affect the effective probe radius, although it could affect the force-indentation curve in the low load/small indentation region.

### *Force Constants*

In figure S10, we have compiled the force constants from various colloidal probes used in this study. For each cantilever, we plot the force constant as measured by the manufacturer with a vibrometer, and as determined by a thermal tune recorded in an AFM. As it can be seen, the measured force constants can deviate by as much as 20 % from the manufacturer's value. The manufacturer's values have been corrected for cantilever tilt in the AFM. Since cantilevers are mounted under a small angle (10˚-12˚ in most instruments) in AFM the effective force constant is only a component of the true force constant (see supporting information 3, effect of cantilever

tilt). In a vibrometer the true amplitude of a vibrating cantilever is measured and thus the true force constant is determined. A thermal tune in an AFM will only measure the component of the true vibration of the cantilever, which is perpendicular to the sample. This component is usually called deflection. Analysing a thermal of the deflection signal will result in the effective force constant as used in AFM. So, here cantilever tilt has not to be considered. However, when using a cantilever in an AFM, we need to correct the true force constant, as measured by a vibrometer accordingly.

The most likely explanation for errors in determining the force constant is errors in calibrating the deflection signal. This hypothesis could only be tested strictly when testing the deflection calibration independently, e.g. with a vibrometer built in an AFM. Since this option is not accessible to us, the only proof of this idea will be the performance of mechanical measurements as discussed below.

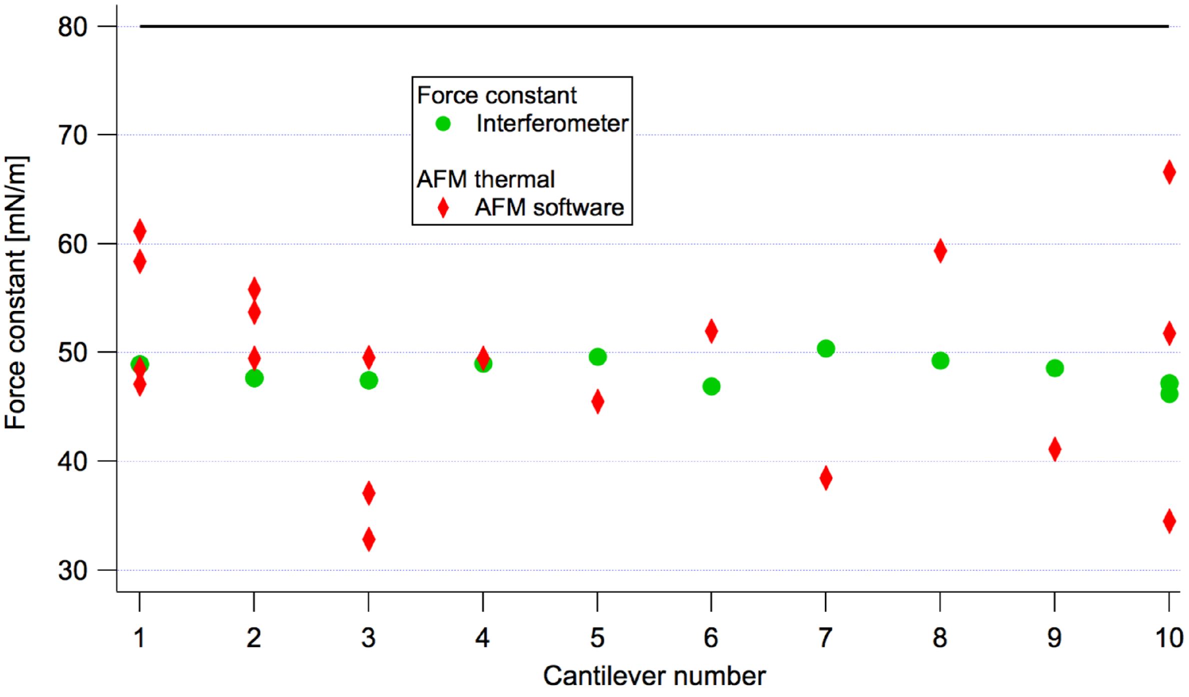


**Figure S10**
Force constants of several colloidal probe cantilevers as measured by the manufacturer using a vibrometer and measured by a thermal tune in air by AFM. The nominal force constant of the manufacturer was 80 mN/m (CP-PNP-SiO-C-5, NanoAndMore, Karlsruhe, Germany) (black horizontal line).

*Deflection Signal*

Since we do not have an experimental way to test or access errors in deflection calibration directly, we can only estimate them based on the following hypothesis: if the main error source while determining force constants by thermals in an AFM is an error in setting the deflection sensitivity, we can use the error in the force constant by AFM in comparison to the exact value determined by a vibrometer to estimate the error in the deflection signal. Since the force constant, as determined by a thermal is inversely proportional to the deflection signal, an error of say + x% in the force constant, could be explained by an error of -0.5 * x % in the deflection signal.

*Accuracy of the Hertz fitting procedure - contact point*

We have calculated a theoretical force curve for a sample with an elastic modulus of 10 kPa. To the deflection signal we added Gaussian noise of 0.5 nm and 2 nm amplitude. The former is a reasonable value for the thermal noise we expect from the cantilevers used here. The latter may serve as a worst-case scenario, which stands for a real instrument, where in addition to thermal noise other noise sources may also be present. The simulated force curve data were then analysed with the standard procedure described above, where only some slice of the data has been analysed. When using small windows (50 nm, 100 nm, 200 nm), errors in elastic modulus can be very large (3 %). For large window sizes (300 nm or 400 nm), especially when starting the analysis right at the contact point, elastic moduli are reproduced with 0.2 % of the nominal value (see figure S11).

The effect of noise on the accuracy in determining the exact elastic modulus value is mainly due to misjudging of the contact point. As can be seen in figure S11, the error in elastic modulus is highly correlated with the error in the contact point.

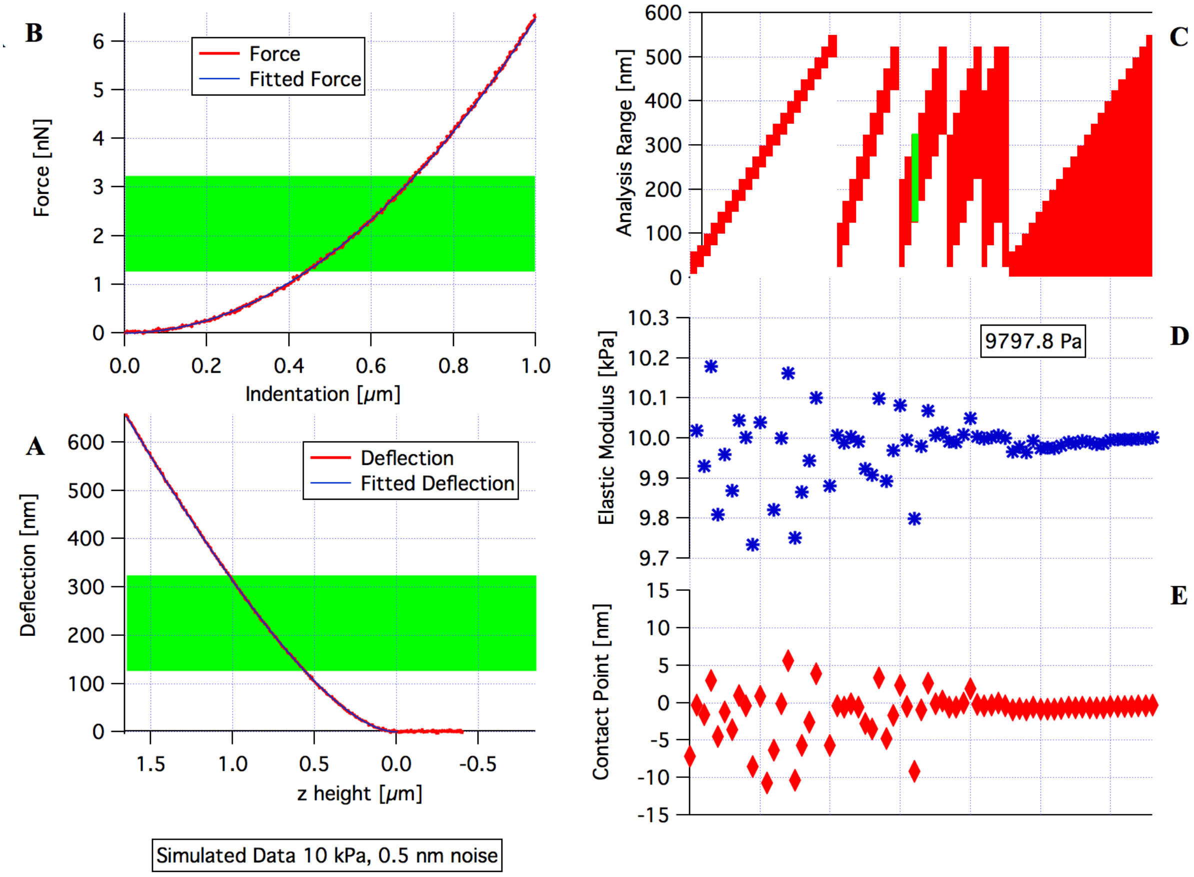


**Figure S11**
Test of the Hertz fit with simulated data (elastic modulus 10 kPa), where 0.5 nm Gaussian noise has been added to the deflection signal. Panel A shows the simulated force curve, Panel B the force versus indentation data calculated from the data of panel A. These data are fitted with the Hertz model in the range indicated by the green bars in panel A and B. When trying out different fit ranges (see bars in panel C) we will receive different Young's moduli values (panel B) and contact points (panel C) as results from the fitting procedure. Numbers in D and E represent the result obtained within the green marked fit range in A, B, and C. If the fit range extends over a large range of data, best starting at zero deflection the results will be very close to the right value (10 kPa). For smaller fit ranges there will be larger deviations, up to 3% in Young's modulus and 10 nm in contact point. Both errors are highly correlated, demonstrating that judging the contact point in the fitting process is actually the most crucial point.
Fig S11
In the above test the main reason for deviations of the calculated elastic modulus from the nominal one (10 kPa) is a misjudgement of the contact point by the noise of the data. The deviation in elastic modulus from the nominal value correlates strongly with the error in determining the contact point.

## *Error Propagation*

Let's assume for the simplicity of the argument here that the main error source is a wrong calibration of the deflection sensitivity. This may be only on the order of a few per cent (see supporting information 2), but to be on the safe side, we may say typical errors here are somewhere between 5 % and 10 %. The degree of this error will depend on the cantilevers used and on the sample and on cleanliness of tip and sample. Since we are interested here in soft samples, cells actually, we tend to use very soft cantilevers, which are very prone to lateral

bending due to friction. And since we are dealing with cells, surface contamination will be inevitable due to the large amount of organic molecules dissolved in culture medium, which will adsorb to sample and tip. So, from our experience with the samples and cantilevers used in this study, we expect typical errors on the order of 5 % or even more in the deflection sensitivity. How will this error propagate and affect our calculated elastic moduli based on the analysis procedure.

Let's first rewrite the basic Hertz model equation with the measurable quantities in an AFM experiment. We will discuss here only the model for a parabolic tip; a similar argument can be employed for four-sided pyramidal tips. Eq. S9 (in supporting information 1) can be written as:

$$E = \frac{F}{\frac{4}{3}*\frac{1}{1-\nu^2}*\sqrt{R}*\delta^{3/2}} = \frac{k_c*(d-d_0)}{\frac{4}{3}*\frac{1}{1-\nu^2}*\sqrt{R}*\delta^{3/2}} = \frac{3}{4}*(1-\nu^2)*\frac{k_c*(d-d_0)}{\sqrt{R}*\delta^{3/2}} \quad \text{(S49)}$$

If we neglect the offset $z_0$ and $d_0$ here, and replace the indentation by the measurable quantities z and d, we will get:

$$E = \frac{3}{4}*(1-\nu^2)*\frac{k_c*d}{\sqrt{R}*(z-d)^{3/2}} \quad \text{(S50)}$$

Let's assume that main error source is in an error in the deflection signal $d^e$, because the deflection sensitivity has been calibrated wrong by a factor c. The true deflection signal shall be denoted by d, whereas the measured or erroneous deflection signal $d^e$ is given by c * d. So, our analysis will result in an erroneous elastic modulus $E^e$, which will be a function of the error factor c:

$$E^e = \frac{3}{4}*(1-\nu^2)*\frac{k_c*d^e}{\sqrt{R}*(z-d^e)^{3/2}} \quad \text{(S51)}$$

$$E = \frac{3}{4}*(1-\nu^2)*\frac{k_c*c*d}{\sqrt{R}*(z-c*d)^{3/2}} \quad \text{(S52)}$$

So, the error in E will be at least proportional to c (through calculation of the force as force constant times deflection). The error through calculation of the indentation (denominator of eq. S52) is harder to quantify, since it depends on the ratio between the z height and the deflection d. In cells measurements, usually the slope of the force curve is very small (0.1 or even smaller), so the z height is much larger than the deflection. Errors in the deflection signal will propagate only partially in the indentation values. For simplifying the argument here, we will neglect this path of error propagation for now.

**Supporting Information 8: The SNAP protocol sheet**

We report here a protocol sheet that can be followed to apply SNAP step by step:

1. Calculate of the correction factor k
2. Calibrate the deflection sensitivity in buffer on glass
3. Record a first AFM thermal spectrum in buffer above glass
4. Change instrument parameters according to the standardized AFM protocol
5. Record a second force curve and thermal as consistency check
6. Record data on your sample

**1. Calculate of the correction factor k**

Cantilever name ____________________

Spring constant determined with a vibrometer $k_{vibrometer}$ : ____________________

Cantilever tilt of your instrument ($\alpha$): ___

Calculate the effective (tilt corrected) force constant $k_{TiltCorrected}$ using the following equation:

$$k_{TiltCorrected} = k_{vibrometer} * \frac{1}{cos^2\alpha}$$

effective (tilt corrected) force constant $k_{TiltCorrected}$: ____________________ 1

Set the κ factor in your software to a value of 1.1. This number may be called amplitude sensitivity correction or similar.

κ factor: ____________1.1_________ 2

**2. Calibrate the Deflection Sensitivity in buffer on glass**

The calibration of the deflection sensitivity needs to be done on a stiff substrate (e.g. a clean glass slide).

Mount your probe and the sample in the AFM. Add the liquid, adjust the laser beam to the cantilever probe and the photo detector position. Engage the tip to the sample and possibly readjust at this point laser diode and detector. Do not adjust laser diode or photo detector any more beyond this point. If you are forced to do it within the subsequent tasks, you need to restart here.

*Force curve for determining deflection sensitivity*

Record a force curve (at 1µm scan size, 1 Hz force rate, 2 µm/s scan speed, trigger threshold/maximum deflection 150 nm) and adjust the cantilever sensitivity with your instrument by fitting a line in a suitable data range (e.g. 25..125 nm deflection).

deflection sensitivity: ____________ 3

force constant: ____________ mN/m

**3. Record a first AFM thermal spectrum in buffer above glass**

Withdraw your AFM tip from the surface by at least 20 µm.

Record a thermal spectrum averaging data at least over 20 seconds (try to reduce acoustic noise to a minimum).

Analyse the spectrum in your instrument and in the online JAVA applet (http://www.biophysik.uni-bremen.de/start/radmacher-group/data-analysis/hertzfit/). Use always the Lorentzian fit both in the Java applet and in the software of your instruments (if it gives you the possibility to choose).

$k_{th}^{Inst}$ by instrument: ____________

$k_{th}^{Java}$ by JAVA applet (Lorentzian) ____________ 4

**4. Change instrument parameters according to the Standardized AFM protocol**

Change the deflection sensitivity by multiplying the old value with the correction factor λ.

λ factor is based on the $k_{th}^{Java}$ 4 from Applet and $k_{TiltCorrected}$ 1 from above.

$$\lambda = \sqrt{\frac{k_{th}{}^{JAVA}}{k_{TiltCorrected}}}$$

λ: ____________

Multiply the deflection sensitivity from 3 by λ to get the new corrected deflection sensitivity and enter it in your instrument's software: ____________ 5

Enter $k_{tiltcorrected}$ from 1 as spring constant in your AFM.

Do not change the deflection sensitivity anymore.

**5. Record a second force curve and thermal as consistency check**

Just for validation of the process record another force curve and a thermal without changing any parameter.

Read the following values from your instrument:

deflection sensitivity: ____________

force constant: ____________

κ factor: ____________

These values shall be identical to the ones from 5 1 and 2.

**6. Record data on your sample**

At this point you can proceed acquiring data on your sample without changing parameters anymore.

# Supporting Information 9: Typical Force Data on Gels

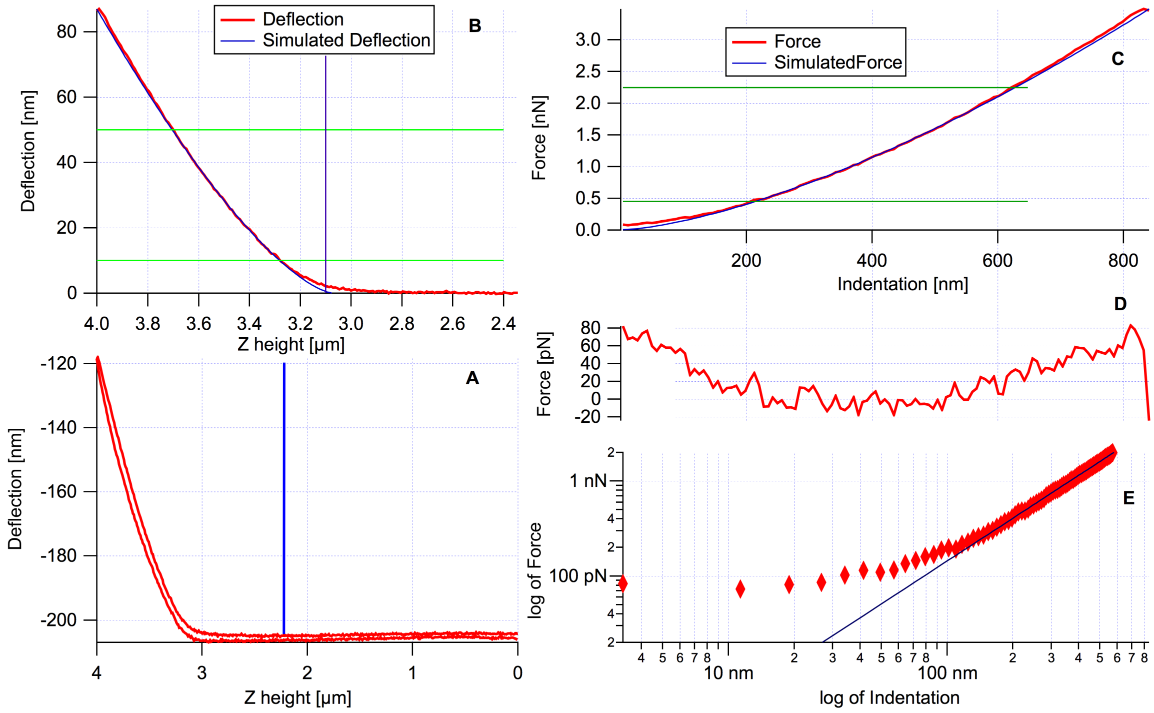


**Figure S12**

Typical force curve on a gel. A shows the raw deflection data, B is the curve of interest (approach) after subtracting the deflection offset and showing in blue the simulated curve, which have been obtained by a Hertz fit. The vertical blue line denotes the contact point, which has been obtained during the Hertz fit. The horizontal green lines show the range of deflection data used for the fit. Panel C shows the loading force versus indentation data as calculated from the force curve data together with the corresponding data from the Hertz fit (blue line). Panel D shows the residuals (i.e. the simulated data versus measured data). Except at very low indentation no systematic variations can be seen. Panel E shows the loading force versus indentation on a log scale (same data as in panel C). Except at very low forces (which correspond to the noise level of the cantilever/instrument) the data lie on a line, which shows that the Hertz model describes the data adequately.

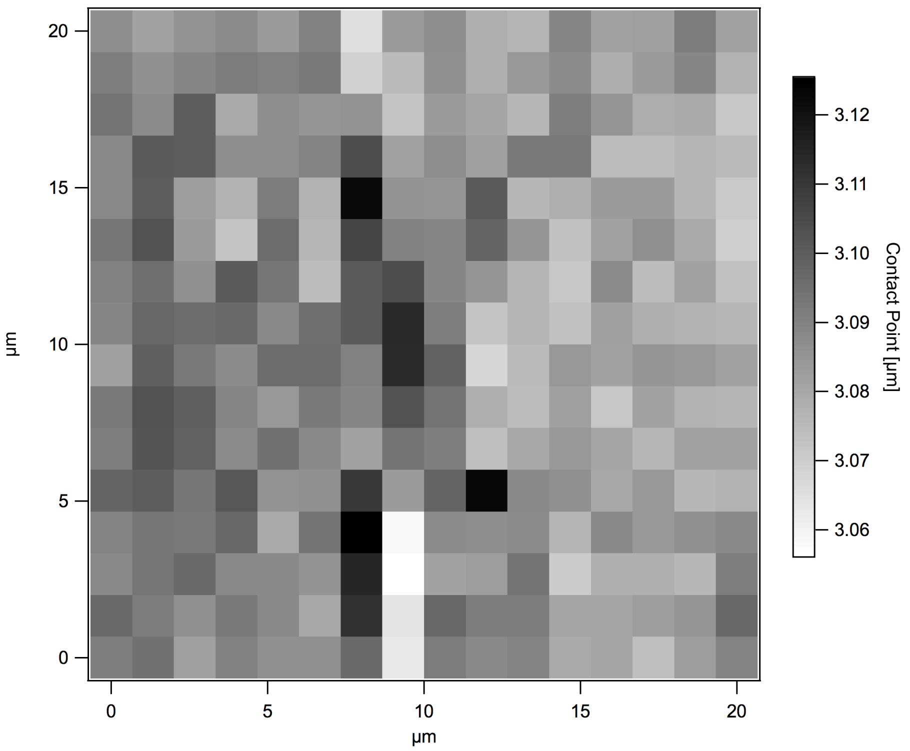


**Figure S13**
Sample height (using the contact point from the Hertz fit) on a gel.

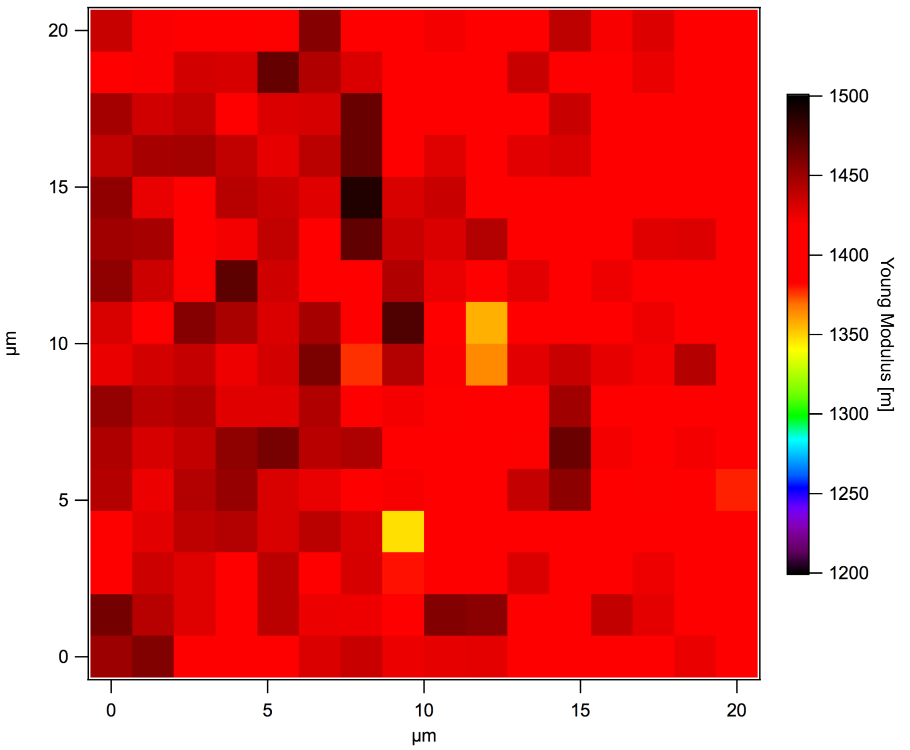


**Figure S14**
Young's modulus as calculated by the Hertz fit on a gel.

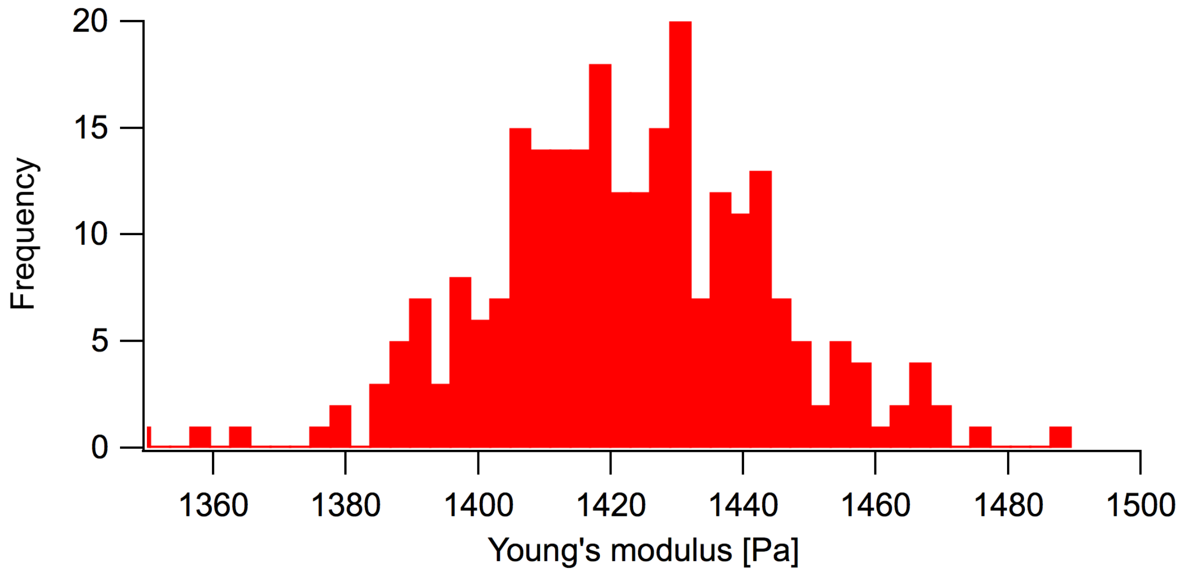


**Figure S15**
Histogram of Young's moduli presented in fig S17. This force map shows a rather narrow distribution with a well defined peak. We choose as a descriptor of the Young's modulus the median, since the distribution is asymmetric which will lead to systematic errors using the mean values.

# Supporting Information 10: Typical Force Data on Cells

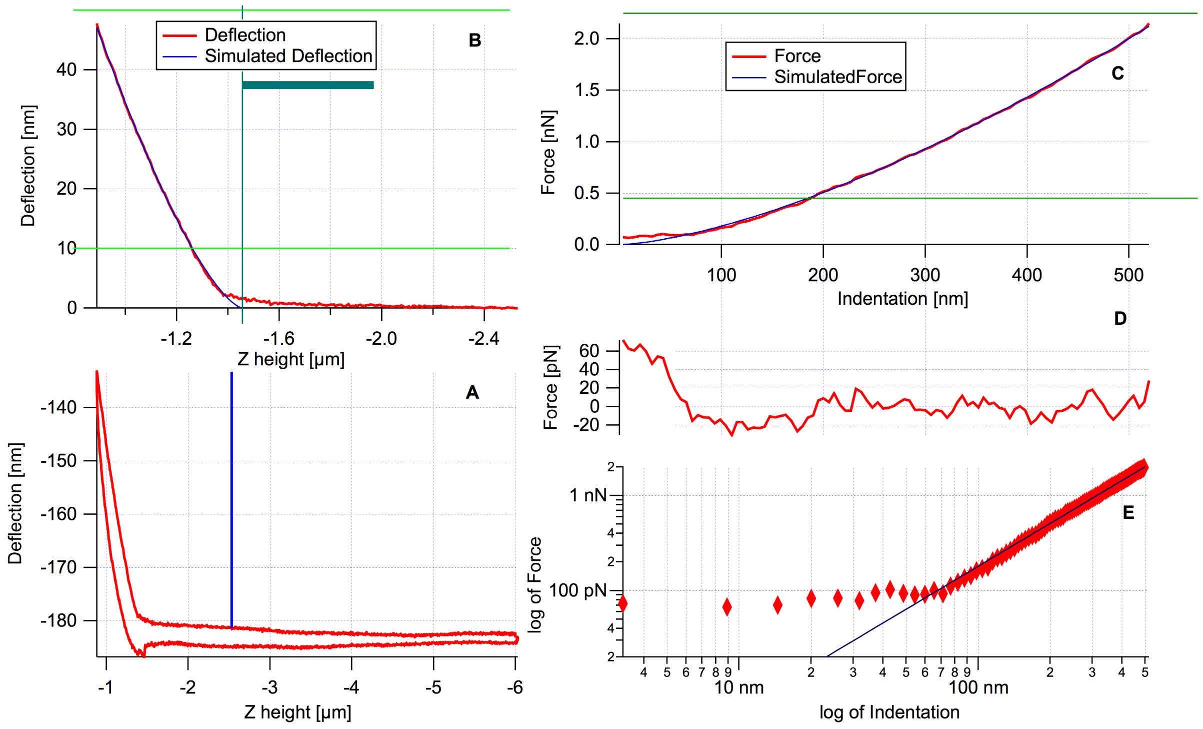


**Figure S16**

Typical force curve on a cell. A shows the raw deflection data, B is the curve of interest (approach) after subtracting the deflection offset and showing in blue the simulated curve, which have been obtained by a Hertz fit. The vertical blue line denotes the contact point, which has been obtained during the Hertz fit. The horizontal green lines show the range of deflection data used for the fit. Panel C shows the loading force versus indentation data as calculated from the force curve data together with the corresponding data from the Hertz fit (blue line). Panel D shows the residuals (i.e. the simulated data versus measured data). Except at very low indentation no systematic variations can be seen. Panel E shows the loading force versus indentation on a log scale (same data as in panel C). Except at very low forces (which correspond to the noise level of the cantilever/instrument) the data lie on a line, which shows that the Hertz model describes the data adequately.

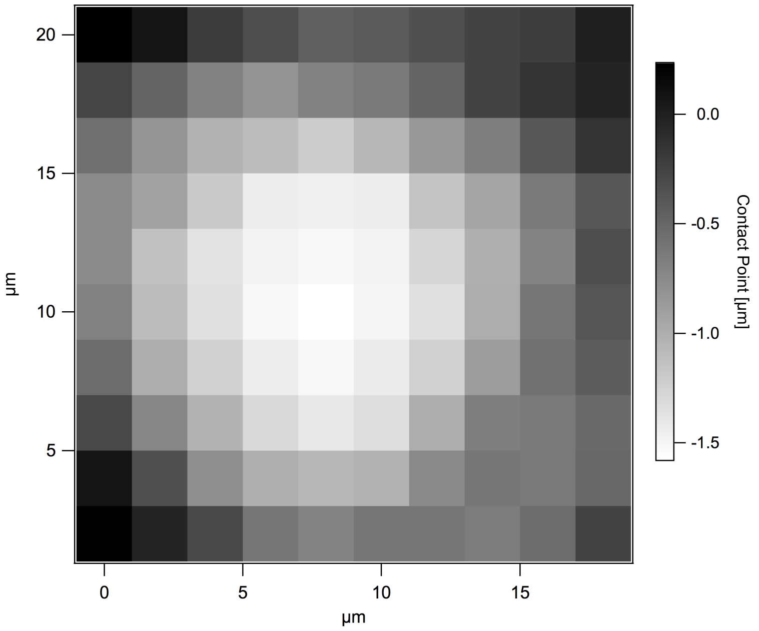


**Figure S17**
Sample height (using the contact point from the Hertz fit) shows that this force map was centered around one cell, but also covers the periphery, i.e. the junctions to the neighboring cells.

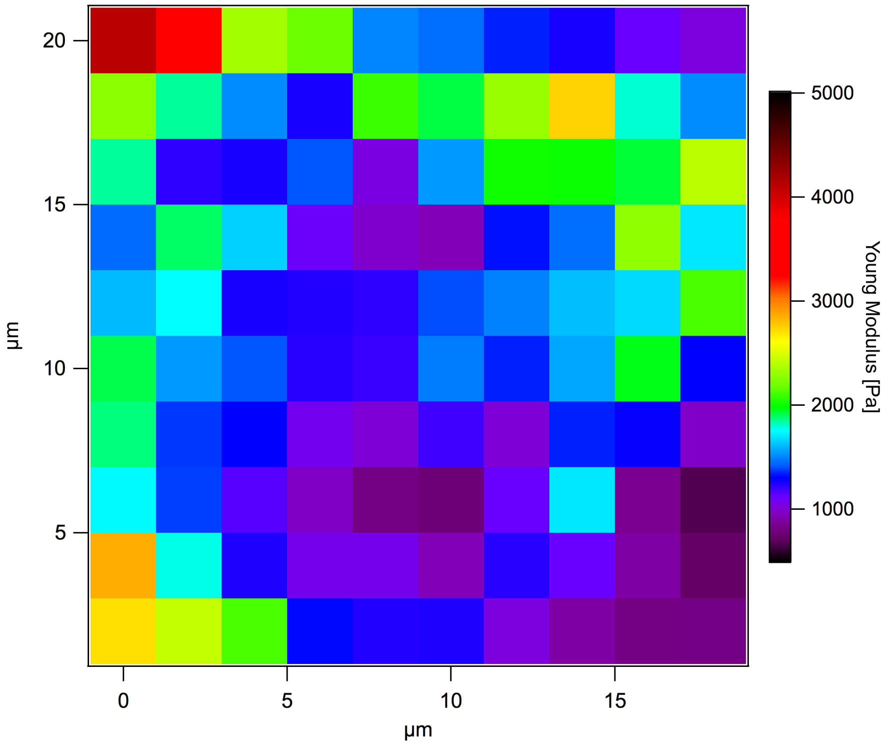


**Figure S18**
Young's modulus as calculated by the Hertz fit. In the center of the cell (see topography in fig S17, the cell is softest and stiffer in the periphery, i.e. the junctions to the neighboring cells.

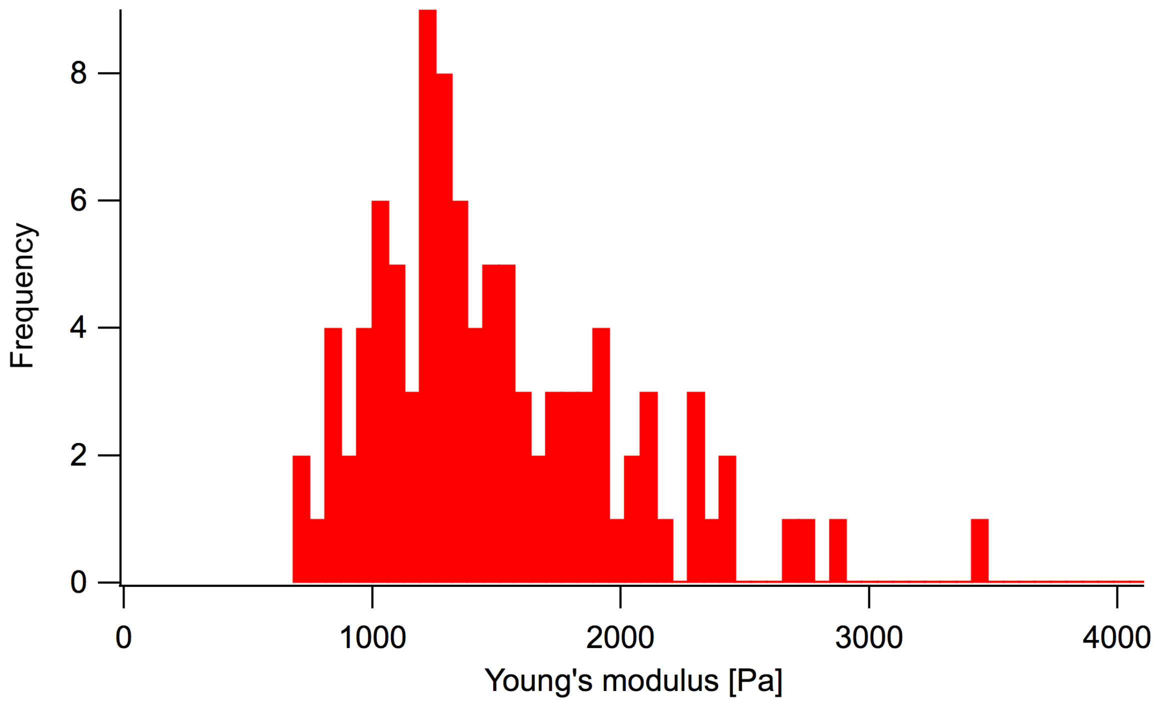


**Figure S19**
Histogram of Young's moduli presented in fig S17. This force map shows a rather narrow distribution (for cells) with a well defined peak. We choose as a descriptor of the cell's Young's modulus the median, since the distribution is asymmetric which will lead to systematic errors using the mean values.

## Supporting Information References

1 Gates, R. S. & Reitsma, M. G. Precise atomic force microscope cantilever spring constant calibration using a reference cantilever array. *Rev. Sci. Instrum.* **78**, 086101 (2007).
2 Gates, R. S., Reitsma, M. G., Kramar, J. A. & Pratt, J. R. Atomic Force Microscopy cantilever flexural stiffness calibration: toward a standard traceable method. *J. Res. NIST* **116**, 703-727 (2011).
3 Cleveland, J. P., Manne, S., Bocek, D. & Hansma, P. K. A nondestructive method for determining the spring constant of cantilevers for scanning force microscopy. *Rev. Sci. Instrum.* **64**, 403-405 (1993).
4 Tortonese, M. & Kirk, M. in *Micromachining and Imaging* 53-60 (IEEE, 1997).
5 Hutter, J. L. & Bechhoefer, J. Calibration of atomic-force microscope tips. *Rev. Sci. Instrum.* **64**, 1868-1873 (1993).
6 Sader, J. E. Parallel beam approximation for V-shaped atomic force microscope cantilevers. *Rev. Sci. Instrum.* **66**, 4583-4587 (1995).
7 Sader, J. E., Larson, I., Mulvaney, P. & White, L. R. Method for the calibration of atomic force microscope cantilevers. *Rev. Sci. Instrum.* **66**, 3789-3797 (1995).
8 Pirzer, T. & Hugel, T. Atomic force microscopy spring constant determination in viscous liquids. *Rev. Sci. Instrum.* **80**, 035110 (2009).
9 Walters, D. A. *et al.* Short cantilevers for atomic force microscopy. *Rev. Sci. Instrum.* **67**, 3583-3590 (1996).
10 Radmacher, M. & Doschke, H. *Hertz model analysis* (2014).
11 Hertz, H. Über die Berührung fester elastischer Körper. *J. Reine Angew. Mathematik* **92**, 156-171 (1882).
12 Sneddon, I. N. The relation between load and penetration in the axisymmetric Boussinesq problem for a punch of arbitrary profile. *Int. J. Eng. Sci.* **3**, 47-57 (1965).
13 Gavara, N. & Chadwick, R. Determination of the elastic moduli of thin samples and adherent cells using conical atomic force tips. *Nat. Nanotechnol.* **7**, 733-736 (2012).
14 Rico, F., Roca-Cusachs, P., Gavara, N., Farré, R., Rotger, M., Navajas, D. Probing mechanical properties of living cells by atomic force microscopy with blunted pyramidal cantilever tips. *Phys. Review E* **72**, 021914 (2005).
15 Bilodeau, G. G. Regular pyramid punch problem. *J. Appl. Mech.* **59**, 519-523 (1992).
16 Barber, J. R. & Billings, D. A. An approximate solution for the contact area and elastic compliance of a smooth punch of arbitrary shape. *Int. J. Mech. Sci.* **32**, 991-997 (1990).
17 Radmacher, M. in *Methods in Cell Biology* (eds Y-l. Wang & D. E. Discher) 347-372 (Academic Press, 2007).
18 Lin, D. C., Dimitriadis, E. K. & Horkay, F. Robust strategies for automated AFM force curve analysis - I. Non-adhesive indentation of soft, inhomogeneous materials. *Trans. ASME* **129**, 430-440 (2007).
19 Crick, S. L. & Yin, F. C. Assessing micromechanical properties of cells with atomic force microscopy: importance of the contact point. *Biomech. Model. Mechanobiol.* **6**, 199-210 (2007).
20 Butt, H.-J. & Jaschke, M. Thermal Noise in Atomic Force Microscopy. N*anotechnology* **6**, 1-7 (1995).
21 Butt, H.-J. & Jaschke, M. Calculation of thermal noise in atomic force microscopy. *Nanotechnology* **6**, 1-7 (1995).

22 Proksch, R., Schäffer, T. E., Cleveland, J. P., Callahan, R. C. & Viani, M. B. Finite optical spot size and position corrections in the spring constant calibration. *Nanotechnology* **4**, 1344-1350 (2004).

23 Hutter, J. Comment on Tilt of Atomic Force Microscope Cantilevers: Effect on Spring Constant and Adhesion Measurements. *Langmuir* **21**, 2630-2632 (2005).

24 Ohler, B. in *Application Note: Practical advice on the determination of cantilever spring constants* (Veeco Instruments, http://nanoscaleworld.bruker-axs.com/nanoscaleworld/media/p/143.aspx, 2007).

25 Ohler, B. Cantilever spring constant calibration using laser Doppler vibrometry. *Rev. Sci. Instrum.* **78**, 063701 (2007).

26 Melcher, J., Hu, S. & Raman, A. Equivalent point-mass models of continous atomic force microscope probes. *Appl. Phys. Lett.* **91**, 053101 (2007).

27 Elson, E. L. Cellular mechanics as an indicator of cytoskeletal structure and function. *Ann. Rev. Biophys. Biophys. Chem.* **17**, 397-430 (1988).

28 Rico, F., Roca-Cusachs, P., Sunyer, R., Farré, R. & Navajas, D. Cell dynamic adhesion and elastic properties probed with cylindrical atomic force microscopy cantilever tips. *J. Mol. Recogn.* **20**, 459-466, (2007).

29 Indrieri, M., Podestà, A., Bongiorno, G., Marchesi, D. & Milani, P. Adhesive-free colloidal probes for nanoscale force measurements: Production and characterization. *Review of Scientific Instruments* **82**, - (2011).

30 Luca Puricelli, Massimiliano Galluzzi, Carsten Schulte, Alessandro Podestà, and Paolo Milani, Nanomechanical and topographical imaging of living cells by atomic force microscopy with colloidal probes, *Review of Scientific Instruments* 86, 033705 (2015).
